# Supplementary material for: Integrated analysis of the transcriptome-wide m6A methylome in preeclampsia and healthy control placentas
Source: PeerJ. 2020 Sep 15;8:e9880. doi: 10.7717/peerj.9880 (PMC7500358; doi:10.7717/peerj.9880)
Supplement: Supplemental Information 1 [file peerj-08-9880-s001.docx]

**Table S1. RNA quantification and quality assurance by NanoDrop ND-1000.**

| **Group** | **Sample ID** | **OD260/280 Ratio** | **OD260/230 Ratio** | **concentration (ng/μl)** | **Volume (μl)** | **Quantity (ng)** |
| --- | --- | --- | --- | --- | --- | --- |
| **Preeclampsia** | **ECL001** | **1.97** | **2.31** | **960.95** | **140** | **134533.00** |
|  | **ECL003** | **1.97** | **2.27** | **1080.80** | **130** | **140504.00** |
|  | **ECL004** | **1.98** | **2.33** | **1315.30** | **100** | **131530.00** |
|  | **ECL005** | **2.00** | **2.28** | **1744.70** | **100** | **174470.00** |
| **Control** | **NEG002** | **1.98** | **2.34** | **1132.57** | **100** | **113257.00** |
|  | **NEG003** | **1.98** | **2.34** | **1301.77** | **90** | **117159.30** |
|  | **NEG004** | **1.97** | **2.26** | **971.00** | **110** | **106810.00** |
|  | **NEG005** | **1.98** | **2.31** | **1366.48** | **90** | **122983.20** |

**For spectrophotometer, the O.D. A260 /A280 ratio should be close to 2.0 for pure RNA (ratios between 1.8 and 2.1 are acceptable). The O.D. A260/A230 ratio should be more than 1.8.**
