# Supplementary material for: Integrated analysis of the transcriptome-wide m6A methylome in preeclampsia and healthy control placentas
Source: PeerJ. 2020 Sep 15;8:e9880. doi: 10.7717/peerj.9880 (PMC7500358; doi:10.7717/peerj.9880)
Supplement: Supplemental Information 2 [file peerj-08-9880-s002.docx]

**Table S2. Quality control of m6A-RIP-seq and RNA-seq.**

| **Sample** | **m6A-RIP-seq** | | | | | **RNA-Seq** | | |
| --- | --- | --- | --- | --- | --- | --- | --- | --- |
|  | **Group** | **Total reads** | **Q30%** | **Unique aligned(%)** | **All aligned(%)** | **Total reads** | **Q30%** | **Mapped%** |
| **ECL001** | ECL001-Input | 21894803 | 97.51 | 74.96 | 96.43 | 39010006 | 88.26 | 90.51 |
|  | ECL001-IP | 19056620 | 96.55 | 80.96 | 95.18 |  |  |  |
| **ECL003** | ECL003-Input | 21937585 | 97.38 | 70.45 | 96.18 | 40222106 | 87.79 | 89.01 |
|  | ECL003-IP | 22311265 | 96.43 | 77.19 | 94.58 |  |  |  |
| **ECL004** | ECL004- IP | 17676471 | 96.46 | 80.27 | 95.87 | 38919482 | 87.63 | 90.26 |
|  | ECL004-Input | 23231496 | 97.5 | 73.17 | 97.28 |  |  |  |
| **ECL005** | ECL005-Input | 20643719 | 97.47 | 73.8 | 97.08 | 35899004 | 88.10 | 90.64 |
|  | ECL005-IP | 22286807 | 96.28 | 80.65 | 95.1 |  |  |  |
| **NEG002** | NEG002-Input | 22302496 | 97.43 | 73.51 | 96.3 | 39166770 | 88.39 | 90.53 |
|  | NEG002-IP | 20443403 | 96.53 | 77.96 | 94.47 |  |  |  |
| **NEG003** | NEG003-Input | 18189639 | 97.52 | 73.39 | 96.9 | 33961246 | 87.81 | 90.38 |
|  | NEG003-IP | 21780808 | 96.47 | 78.61 | 95.45 |  |  |  |
| **NEG004** | NEG004-Input | 21263935 | 97.49 | 73.73 | 97.05 | 44123532 | 88.02 | 90.63 |
|  | NEG004-IP | 22039967 | 96.46 | 80.95 | 95.24 |  |  |  |
| **NEG005** | NEG005-Input | 21935007 | 97.52 | 74.52 | 97.23 | 38736314 | 87.29 | 90.00 |
|  | NEG005-IP | 23688316 | 96.56 | 81.52 | 95.41 |  |  |  |
