# Supplementary material for: Integrated analysis of the transcriptome-wide m6A methylome in preeclampsia and healthy control placentas
Source: PeerJ. 2020 Sep 15;8:e9880. doi: 10.7717/peerj.9880 (PMC7500358; doi:10.7717/peerj.9880)
Supplement: Supplemental Information 3 [file peerj-08-9880-s003.docx]

**Table S3. All differently expressed m6A peaks in preeclampsia.**

| **Gene name** | **Transcript ID** | **Regulation** | **Fold change** | **Chromosome** | **Peak start** | **Peak end** | **Peak region** | ***p* value** |
| --- | --- | --- | --- | --- | --- | --- | --- | --- |
| *HSPA1A* | ENST00000441618.1 | Up | 47.50 | GL000251.1 | 3294734 | 3294974 | cds | 5.13E-06 |
| *DMWD* | ENST00000598237.1 | Up | 18.38 | chr19 | 46293970 | 46294029 | cds, utr3 | 1.82E-02 |
| *HYOU1* | ENST00000572361.1 | Up | 15.67 | JH159138.1 | 67181 | 68226 | cds, utr3 | 5.62E-03 |
| *BRCA1* | ENST00000471181.2 | Up | 15.14 | chr17 | 41245740 | 41245861 | cds | 6.61E-03 |
| *NDUFB2* | ENST00000472695.1 | Up | 13.36 | chr7 | 140396821 | 140400705 | utr5 | 4.79E-02 |
| *SLC39A1* | ENST00000413622.1 | Up | 13.09 | chr1 | 153939949 | 153940038 | utr5 | 1.38E-02 |
| *HYOU1* | ENST00000572084.2 | Up | 13.00 | JH159138.1 | 67162 | 68237 | cds, utr3 | 7.94E-03 |
| *SCAF11* | ENST00000465950.1 | Up | 13.00 | chr12 | 46322790 | 46322910 | utr5 | 2.75E-02 |
| *SLC25A29* | ENST00000554912.1 | Up | 12.47 | chr14 | 100760163 | 100760343 | utr5 | 2.63E-02 |
| *MID1IP1* | ENST00000336949.6 | Up | 9.99 | chrX | 38663134 | 38663344 | utr5 | 1.55E-02 |
| *DSCAM* | ENST00000400454.1 | Up | 9.85 | chr21 | 41384898 | 41385109 | utr3, cds | 2.29E-02 |
| *DSCAM* | ENST00000404019.2 | Up | 9.71 | chr21 | 41384917 | 41385156 | utr3, cds | 2.29E-02 |
| *MPP6* | ENST00000396475.2 | Up | 8.69 | chr7 | 24613173 | 24659811 | utr5 | 4.90E-02 |
| *ATAD5* | ENST00000321990.4 | Up | 8.51 | chr17 | 29222496 | 29222797 | utr3 | 3.24E-02 |
| *FMR1* | ENST00000602057.1 | Up | 8.51 | JH806600.2 | 3474351 | 3474501 | utr3 | 3.02E-02 |
| *FMR1* | ENST00000600585.1 | Up | 8.51 | JH806600.2 | 3474348 | 3474499 | utr3 | 3.02E-02 |
| *MPP6* | ENST00000432190.1 | Up | 8.34 | chr7 | 24613124 | 24663402 | cds, utr5 | 3.72E-02 |
| *DLK2* | ENST00000357338.3 | Up | 7.36 | chr6 | 43422544 | 43423891 | cds, utr5 | 2.40E-03 |
| *TUBA8* | ENST00000608634.1 | Up | 7.21 | chr22 | 18628492 | 18628582 | cds | 4.37E-02 |
| *DLK2* | ENST00000414245.1 | Up | 7.11 | chr6 | 43422550 | 43423865 | cds, utr5 | 3.63E-03 |
| *SZT2* | ENST00000310739.4 | Up | 6.96 | chr1 | 43872115 | 43872325 | utr3 | 1.02E-02 |
| *DCLK3* | ENST00000416516.2 | Up | 6.96 | chr3 | 36779118 | 36779566 | cds | 4.07E-02 |
| *SZT2* | ENST00000372450.4 | Up | 6.92 | chr1 | 43872118 | 43872327 | utr3 | 1.00E-02 |
| *DLK2* | ENST00000372488.3 | Up | 6.19 | chr6 | 43422543 | 43423268 | cds, utr5 | 9.55E-03 |
| *DLK2* | ENST00000372485.1 | Up | 5.98 | chr6 | 43422546 | 43423269 | cds, utr5 | 1.10E-02 |
| *BRCA1* | ENST00000493795.1 | Up | 5.70 | chr17 | 41245727 | 41245877 | cds | 2.09E-02 |
| *CCDC89* | ENST00000316398.3 | Up | 5.70 | chr11 | 85396120 | 85396721 | cds | 1.38E-02 |
| *FGF18* | ENST00000274625.5 | Up | 5.50 | chr5 | 170883915 | 170884390 | utr3 | 5.89E-03 |
| *SPIRE1* | ENST00000410092.3 | Up | 5.35 | chr18 | 12657714 | 12657865 | cds | 2.88E-02 |
| *SPIRE1* | ENST00000409402.4 | Up | 5.35 | chr18 | 12657712 | 12657863 | cds | 2.88E-02 |
| *FAM134B* | ENST00000306320.9 | Up | 4.99 | chr5 | 16616866 | 16617077 | cds | 4.07E-02 |
| *BRCA1* | ENST00000470026.1 | Up | 4.76 | chr17 | 41245689 | 41245868 | cds | 2.24E-02 |
| *MYL5* | ENST00000506838.1 | Up | 4.72 | chr4 | 668950 | 669130 | utr5 | 8.32E-03 |
| *TUBA8* | ENST00000330423.3 | Up | 4.63 | chr22 | 18613785 | 18613934 | utr3, cds | 1.86E-02 |
| *CHST1* | ENST00000308064.2 | Up | 4.56 | chr11 | 45670993 | 45671412 | cds, utr3 | 6.61E-03 |
| *BRCA1* | ENST00000477152.1 | Up | 4.38 | chr17 | 41245692 | 41245873 | cds | 2.95E-02 |
| *ZNF35* | ENST00000453164.1 | Up | 4.35 | chr3 | 44700241 | 44700387 | utr3, cds | 4.27E-02 |
| *ZNF774* | ENST00000354377.3 | Up | 4.23 | chr15 | 90903685 | 90904045 | cds | 5.89E-03 |
| *SH3BGR* | ENST00000423596.1 | Up | 4.17 | chr21 | 40871748 | 40883702 | utr3, cds | 1.70E-02 |
| *ATAD5* | ENST00000321990.4 | Up | 4.14 | chr17 | 29161321 | 29161562 | cds | 2.04E-02 |
| *DHDDS* | ENST00000530781.1 | Up | 4.08 | chr1 | 26759039 | 26759382 | utr5 | 1.00E-02 |
| *MAP3K9* | ENST00000554752.2 | Up | 4.08 | chr14 | 71275558 | 71275888 | cds | 1.58E-03 |
| *BRCA1* | ENST00000357654.3 | Up | 4.03 | chr17 | 41245690 | 41245870 | cds | 2.75E-02 |
| *MAP3K9* | ENST00000555993.2 | Up | 3.84 | chr14 | 71275563 | 71275893 | utr5, cds | 2.14E-03 |
| *AATK* | ENST00000417379.1 | Up | 3.81 | chr17 | 79095231 | 79095531 | cds | 6.46E-03 |
| *ZNF724P* | ENST00000418100.1 | Up | 3.76 | chr19 | 23404638 | 23405411 | utr3, cds | 1.10E-03 |
| *MAP3K9* | ENST00000381250.4 | Up | 3.66 | chr14 | 71275566 | 71275895 | utr5, cds | 3.31E-03 |
| *SLAMF7* | ENST00000368042.3 | Up | 3.48 | chr1 | 160723623 | 160723923 | utr3 | 3.24E-02 |
| *ZZZ3* | ENST00000433749.1 | Up | 3.48 | chr1 | 78098923 | 78099010 | cds | 4.90E-02 |
| *PAQR8* | ENST00000442253.2 | Up | 3.48 | chr6 | 52270805 | 52270956 | utr3 | 3.55E-02 |
| *KIAA1731* | ENST00000325212.6 | Up | 3.43 | chr11 | 93400891 | 93402936 | cds | 3.98E-04 |
| *SLAMF7* | ENST00000359331.4 | Up | 3.41 | chr1 | 160723628 | 160723927 | utr3 | 3.55E-02 |
| *FGD1* | ENST00000375135.3 | Up | 3.34 | chrX | 54522150 | 54522360 | utr5 | 3.55E-02 |
| *ARSK* | ENST00000380009.4 | Up | 3.34 | chr5 | 94940258 | 94940349 | utr3 | 3.31E-02 |
| *AATK* | ENST00000326724.4 | Up | 3.32 | chr17 | 79095221 | 79095550 | cds | 1.05E-02 |
| *KCNMB3* | ENST00000314235.5 | Up | 3.32 | chr3 | 178969016 | 178969403 | utr5 | 1.70E-02 |
| *DIO2* | ENST00000557125.1 | Up | 3.20 | chr14 | 80668989 | 80669219 | utr3 | 4.90E-03 |
| *KIAA1731* | ENST00000344196.4 | Up | 3.10 | chr11 | 93400872 | 93408746 | utr5 | 5.13E-04 |
| *VPS13A* | ENST00000360280.3 | Up | 3.07 | chr9 | 80030940 | 80031061 | utr3 | 3.16E-02 |
| *KIAA1731* | ENST00000411936.1 | Up | 3.05 | chr11 | 93400872 | 93408746 | cds | 6.17E-04 |
| *KCNMB3* | ENST00000392685.2 | Up | 3.05 | chr3 | 178969030 | 178969418 | utr5 | 2.75E-02 |
| *ZFP36L2* | ENST00000282388.3 | Up | 3.03 | chr2 | 43451983 | 43452133 | cds | 3.47E-03 |
| *DIO2* | ENST00000555750.1 | Up | 3.01 | chr14 | 80668970 | 80669240 | utr3, cds | 5.50E-03 |
| *ROM1* | ENST00000278833.3 | Up | 2.99 | chr11 | 62382382 | 62382592 | utr3 | 2.40E-02 |
| *DNAJA4* | ENST00000394852.3 | Up | 2.91 | chr15 | 78556975 | 78562846 | utr5, cds | 4.57E-04 |
| *RGS12* | ENST00000336727.3 | Up | 2.91 | chr4 | 3318793 | 3318974 | cds | 1.70E-02 |
| *VPS13A* | ENST00000484581.2 | Up | 2.89 | chr9 | 80030953 | 80031072 | utr3 | 4.27E-02 |
| *REL* | ENST00000295025.8 | Up | 2.85 | chr2 | 61121657 | 61128193 | cds | 3.89E-02 |
| *ROM1* | ENST00000534093.1 | Up | 2.83 | chr11 | 62382393 | 62382572 | utr3 | 3.80E-02 |
| *CREBBP* | ENST00000262367.5 | Up | 2.77 | chr16 | 3930128 | 3930398 | utr5 | 2.34E-02 |
| *GLCCI1* | ENST00000223145.5 | Up | 2.69 | chr7 | 8009110 | 8009290 | cds | 2.29E-02 |
| *RGS12* | ENST00000382788.3 | Up | 2.66 | chr4 | 3318782 | 3318963 | cds | 2.45E-02 |
| *HOOK1* | ENST00000371208.3 | Up | 2.66 | chr1 | 60331574 | 60336781 | cds | 2.57E-03 |
| *HOOK1* | ENST00000395561.2 | Up | 2.62 | chr1 | 60331571 | 60336778 | cds | 2.95E-03 |
| *TBC1D1* | ENST00000508802.1 | Up | 2.57 | chr4 | 37903868 | 37904108 | cds | 1.55E-02 |
| *RPUSD1* | ENST00000565377.1 | Up | 2.55 | chr16 | 835982 | 836130 | cds | 1.78E-02 |
| *GPR65* | ENST00000267549.3 | Up | 2.55 | chr14 | 88476871 | 88477169 | utr5 | 3.31E-02 |
| *DCLRE1B* | ENST00000369563.3 | Up | 2.55 | chr1 | 114454887 | 114455037 | utr3 | 4.37E-02 |
| *RGS12* | ENST00000543385.1 | Up | 2.53 | chr4 | 3318785 | 3318965 | cds | 3.16E-02 |
| *RGS12* | ENST00000344733.5 | Up | 2.53 | chr4 | 3318786 | 3318966 | cds | 3.16E-02 |
| *TBC1D1* | ENST00000402522.1 | Up | 2.51 | chr4 | 37903875 | 37904113 | cds | 1.78E-02 |
| *SLC25A23* | ENST00000301454.4 | Up | 2.46 | chr19 | 6440104 | 6440462 | utr3 | 1.41E-02 |
| *CAPN15* | ENST00000219611.2 | Up | 2.46 | chr16 | 577885 | 586041 | utr5 | 3.31E-02 |
| *ATAD2* | ENST00000521903.1 | Up | 2.46 | chr8 | 124382070 | 124383492 | utr5 | 6.46E-03 |
| *NF2* | ENST00000338641.4 | Up | 2.46 | chr22 | 30091169 | 30091320 | utr3 | 4.47E-02 |
| *NF2* | ENST00000413209.2 | Up | 2.46 | chr22 | 30091168 | 30091319 | utr3 | 4.47E-02 |
| *UACA* | ENST00000322954.6 | Up | 2.45 | chr15 | 70946952 | 70947222 | utr3 | 7.94E-03 |
| *TBC1D1* | ENST00000261439.4 | Up | 2.45 | chr4 | 37903871 | 37904112 | cds | 2.09E-02 |
| *MASTL* | ENST00000375946.4 | Up | 2.45 | chr10 | 27444231 | 27444471 | utr5, cds | 1.51E-02 |
| *CCDC88A* | ENST00000436346.1 | Up | 2.41 | chr2 | 55566685 | 55571559 | cds | 2.34E-03 |
| *VPS13A* | ENST00000376636.3 | Up | 2.38 | chr9 | 80022522 | 80031081 | utr3, cds | 2.45E-02 |
| *TSC22D1* | ENST00000458659.2 | Up | 2.36 | chr13 | 45150221 | 45150372 | utr5 | 4.47E-02 |
| *RPUSD1* | ENST00000569601.1 | Up | 2.35 | chr16 | 835967 | 836145 | cds | 1.66E-02 |
| *METTL4* | ENST00000319888.6 | Up | 2.35 | chr18 | 2567418 | 2571151 | utr5 | 2.45E-02 |
| *ATAD2* | ENST00000287394.5 | Up | 2.35 | chr8 | 124382081 | 124383504 | cds | 8.51E-03 |
| *AXIN2* | ENST00000580513.1 | Up | 2.33 | chr17 | 63554749 | 63557074 | utr5 | 8.13E-03 |
| *HS3ST6* | ENST00000443547.1 | Up | 2.33 | chr16 | 1961463 | 1961847 | utr3, cds | 1.51E-02 |
| *IRAK3* | ENST00000457197.2 | Up | 2.33 | chr12 | 66641644 | 66642123 | utr3, cds | 9.12E-04 |
| *BCOR* | ENST00000378463.1 | Up | 2.33 | chrX | 39921537 | 39922009 | cds | 8.71E-03 |
| *HS3ST6* | ENST00000454677.2 | Up | 2.33 | chr16 | 1961463 | 1961849 | utr3, cds | 1.51E-02 |
| *HS3ST6* | ENST00000293937.3 | Up | 2.33 | chr16 | 1961463 | 1961849 | utr3, cds | 1.51E-02 |
| *CCR7* | ENST00000579344.1 | Up | 2.31 | chr17 | 38710640 | 38710879 | utr3 | 3.39E-02 |
| *LTBP3* | ENST00000322147.4 | Up | 2.31 | chr11 | 65325189 | 65325430 | cds | 5.50E-05 |
| *BCOR* | ENST00000442018.1 | Up | 2.30 | chrX | 39921537 | 39922010 | cds | 9.12E-03 |
| *AXIN2* | ENST00000585045.1 | Up | 2.28 | chr17 | 63554783 | 63556178 | utr5 | 2.24E-02 |
| *BCOR* | ENST00000378444.4 | Up | 2.28 | chrX | 39921519 | 39922022 | cds | 5.89E-03 |
| *NAPEPLD* | ENST00000465647.1 | Up | 2.27 | chr7 | 102743818 | 102755592 | utr3, cds | 2.57E-02 |
| *BCOR* | ENST00000427012.1 | Up | 2.27 | chrX | 39921514 | 39922046 | cds | 2.69E-03 |
| *SIGLEC7* | ENST00000305628.7 | Up | 2.25 | chr19 | 51645743 | 51648212 | cds | 1.95E-02 |
| *TNFRSF10B* | ENST00000276431.4 | Up | 2.25 | chr8 | 22877705 | 22878153 | utr3 | 1.17E-03 |
| *ARID4A* | ENST00000424658.1 | Up | 2.23 | chr14 | 58768338 | 58771708 | cds | 4.47E-02 |
| *SIGLEC7* | ENST00000317643.6 | Up | 2.22 | chr19 | 51645735 | 51647726 | cds | 1.48E-02 |
| *ZNF654* | ENST00000309495.5 | Up | 2.22 | chr3 | 88188313 | 88188553 | cds, utr5 | 1.45E-02 |
| *PRX* | ENST00000324001.7 | Up | 2.20 | chr19 | 40909736 | 40913900 | utr5, cds | 3.24E-02 |
| *BCOR* | ENST00000413905.1 | Up | 2.20 | chrX | 39921531 | 39922033 | cds | 7.41E-03 |
| *EIF4G3* | ENST00000400422.1 | Up | 2.19 | chr1 | 21180149 | 21181540 | cds | 4.17E-02 |
| *SIGLEC7* | ENST00000601682.1 | Up | 2.19 | chr19 | 51645734 | 51647913 | cds | 2.00E-02 |
| *BCOR* | ENST00000342274.4 | Up | 2.17 | chrX | 39921520 | 39922023 | cds | 9.12E-03 |
| *BCOR* | ENST00000378455.4 | Up | 2.17 | chrX | 39921511 | 39922014 | cds | 7.24E-03 |
| *PPP2R5A* | ENST00000261461.2 | Up | 2.16 | chr1 | 212459056 | 212459503 | utr5, cds | 5.13E-05 |
| *TBC1D24* | ENST00000567020.1 | Up | 2.14 | chr16 | 2552505 | 2552985 | utr3 | 2.69E-02 |
| *NEK1* | ENST00000512193.1 | Up | 2.13 | chr4 | 170477189 | 170498090 | cds | 1.35E-02 |
| *BCOR* | ENST00000397354.3 | Up | 2.13 | chrX | 39921514 | 39922017 | cds | 9.12E-03 |
| *ZNF850* | ENST00000591344.1 | Up | 2.11 | chr19 | 37241292 | 37241532 | cds | 3.31E-02 |
| *METTL4* | ENST00000574538.1 | Up | 2.11 | chr18 | 2567424 | 2571157 | utr5 | 4.07E-02 |
| *CHRD* | ENST00000204604.1 | Up | 2.10 | chr3 | 184107437 | 184107587 | utr3 | 4.07E-02 |
| *MKLN1* | ENST00000352689.6 | Up | 2.10 | chr7 | 131172578 | 131172669 | utr3 | 5.50E-03 |
| *CHRD* | ENST00000348986.3 | Up | 2.10 | chr3 | 184107437 | 184107587 | utr3 | 4.07E-02 |
| *SLC22A23* | ENST00000436008.2 | Up | 2.08 | chr6 | 3269195 | 3269346 | utr3 | 1.29E-02 |
| *PRRG2* | ENST00000246794.5 | Up | 2.06 | chr19 | 50094122 | 50094272 | utr3 | 2.69E-02 |
| *OXSM* | ENST00000280701.3 | Up | 2.04 | chr3 | 25835667 | 25836025 | cds, utr3 | 2.34E-04 |
| *PRX* | ENST00000291825.7 | Up | 2.01 | chr19 | 40909705 | 40913929 | utr5, cds | 2.51E-02 |
| *OXSM* | ENST00000420173.2 | Up | 2.00 | chr3 | 25835636 | 25836025 | cds, utr3 | 2.63E-04 |
| *ZNF10* | ENST00000426665.2 | Up | 1.99 | chr12 | 133732187 | 133732485 | cds | 3.80E-02 |
| *POLH* | ENST00000372236.4 | Up | 1.98 | chr6 | 43581836 | 43582077 | cds | 3.24E-02 |
| *ZFX* | ENST00000539115.1 | Up | 1.97 | chrX | 24231825 | 24232065 | utr3 | 5.01E-04 |
| *ZNF629* | ENST00000262525.4 | Up | 1.96 | chr16 | 30789777 | 30790137 | utr3 | 9.33E-03 |
| *ZBTB7B* | ENST00000368426.3 | Up | 1.96 | chr1 | 154989859 | 154990190 | utr3 | 2.40E-05 |
| *CFLAR* | ENST00000395148.2 | Up | 1.95 | chr2 | 202001348 | 202001498 | utr3 | 6.61E-03 |
| *NKRF* | ENST00000371527.1 | Up | 1.94 | chrX | 118724573 | 118724783 | cds | 4.90E-02 |
| *CBFB* | ENST00000561924.2 | Up | 1.94 | chr16 | 67132626 | 67132685 | utr3, cds | 4.37E-02 |
| *TRIO* | ENST00000344204.4 | Up | 1.92 | chr5 | 14488092 | 14488273 | cds | 1.35E-02 |
| *TRIO* | ENST00000513206.1 | Up | 1.91 | chr5 | 14488093 | 14488274 | cds | 1.38E-02 |
| *TESK1* | ENST00000336395.5 | Up | 1.91 | chr9 | 35605635 | 35605755 | cds | 2.14E-02 |
| *FAM105B* | ENST00000284274.4 | Up | 1.91 | chr5 | 14673760 | 14678873 | cds | 1.66E-02 |
| *DST* | ENST00000518935.1 | Up | 1.91 | chr6 | 56492024 | 56492885 | cds | 4.37E-02 |
| *MECOM* | ENST00000464456.1 | Up | 1.91 | chr3 | 168864626 | 168865015 | utr5 | 9.77E-03 |
| *ANKRD49* | ENST00000544253.1 | Up | 1.90 | chr11 | 94232653 | 94232743 | utr3 | 1.58E-02 |
| *ZFX* | ENST00000379188.3 | Up | 1.90 | chrX | 24231826 | 24232037 | utr3 | 1.35E-03 |
| *PRPF18* | ENST00000378572.3 | Up | 1.90 | chr10 | 13672394 | 13672454 | utr3 | 4.27E-02 |
| *DVL1* | ENST00000378888.5 | Up | 1.89 | chr1 | 1284309 | 1284730 | utr5, cds | 2.88E-04 |
| *ZFX* | ENST00000379177.1 | Up | 1.89 | chrX | 24231836 | 24232017 | utr3 | 2.63E-03 |
| *PTMA* | ENST00000440384.1 | Up | 1.88 | chr2 | 232575255 | 232576093 | cds | 4.57E-02 |
| *KAT6B* | ENST00000372711.1 | Up | 1.88 | chr10 | 76735224 | 76735435 | cds | 4.07E-02 |
| *SLC9A8* | ENST00000541138.1 | Up | 1.88 | chr20 | 48504861 | 48505010 | utr3 | 2.75E-02 |
| *SYBU* | ENST00000532779.1 | Up | 1.87 | chr8 | 110587652 | 110588188 | cds | 2.69E-02 |
| *SYBU* | ENST00000533065.1 | Up | 1.87 | chr8 | 110587652 | 110588188 | cds | 2.69E-02 |
| *NEK1* | ENST00000510533.1 | Up | 1.87 | chr4 | 170477182 | 170498097 | cds | 1.20E-02 |
| *PLEKHA6* | ENST00000272203.3 | Up | 1.87 | chr1 | 204187978 | 204188129 | utr3 | 1.51E-03 |
| *SNTB1* | ENST00000395601.3 | Up | 1.85 | chr8 | 121550700 | 121551059 | utr3 | 8.91E-03 |
| *NEK1* | ENST00000511633.1 | Up | 1.84 | chr4 | 170477204 | 170498090 | cds | 1.82E-02 |
| *C11orf49* | ENST00000536126.1 | Up | 1.84 | chr11 | 47183225 | 47183462 | utr3 | 3.31E-02 |
| *SYBU* | ENST00000424158.2 | Up | 1.83 | chr8 | 110587654 | 110588191 | cds | 3.24E-02 |
| *CDK17* | ENST00000261211.3 | Up | 1.83 | chr12 | 96673832 | 96674660 | utr3, cds | 4.79E-04 |
| *AGTPBP1* | ENST00000357081.3 | Up | 1.83 | chr9 | 88247763 | 88248123 | cds | 3.39E-02 |
| *PLA2G16* | ENST00000323646.5 | Up | 1.83 | chr11 | 63340666 | 63340905 | utr3 | 2.95E-02 |
| *NEK1* | ENST00000439128.2 | Up | 1.83 | chr4 | 170477178 | 170498094 | cds | 1.70E-02 |
| *ENDOG* | ENST00000372642.4 | Up | 1.83 | chr9 | 131584809 | 131584956 | cds, utr3 | 3.80E-03 |
| *STK36* | ENST00000392106.2 | Up | 1.82 | chr2 | 219567014 | 219567344 | utr3 | 1.55E-02 |
| *SYBU* | ENST00000529690.1 | Up | 1.82 | chr8 | 110587650 | 110588213 | cds | 3.02E-02 |
| *NEK1* | ENST00000507142.1 | Up | 1.82 | chr4 | 170477180 | 170498095 | cds | 1.70E-02 |
| *WHSC1* | ENST00000509115.1 | Up | 1.82 | chr4 | 1918717 | 1920116 | cds | 3.98E-03 |
| *DST* | ENST00000244364.6 | Up | 1.82 | chr6 | 56492025 | 56492916 | cds | 4.07E-02 |
| *DST* | ENST00000361203.3 | Up | 1.81 | chr6 | 56492019 | 56492911 | cds | 3.89E-02 |
| *AGTPBP1* | ENST00000432218.1 | Up | 1.81 | chr9 | 88247767 | 88248125 | cds | 3.72E-02 |
| *AAMDC* | ENST00000533193.1 | Up | 1.80 | chr11 | 77583255 | 77583400 | utr3, cds | 3.47E-02 |
| *STK36* | ENST00000295709.3 | Up | 1.79 | chr2 | 219567019 | 219567318 | utr3 | 2.19E-02 |
| *BCAR1* | ENST00000561970.1 | Up | 1.79 | chr16 | 75276970 | 75284786 | utr5 | 9.33E-03 |
| *SYBU* | ENST00000528647.1 | Up | 1.78 | chr8 | 110587642 | 110588211 | cds | 3.63E-02 |
| *GXYLT1* | ENST00000398675.3 | Up | 1.78 | chr12 | 42476276 | 42476577 | utr3 | 1.17E-02 |
| *DST* | ENST00000520645.1 | Up | 1.78 | chr6 | 56492012 | 56492933 | cds | 3.24E-02 |
| *DST* | ENST00000312431.6 | Up | 1.78 | chr6 | 56492012 | 56492934 | cds | 3.24E-02 |
| *DST* | ENST00000370765.6 | Up | 1.78 | chr6 | 56492010 | 56492932 | cds | 3.24E-02 |
| *DST* | ENST00000446842.2 | Up | 1.78 | chr6 | 56492013 | 56492934 | cds | 3.24E-02 |
| *RUSC1* | ENST00000368354.3 | Up | 1.78 | chr1 | 155300393 | 155300544 | utr3 | 6.46E-03 |
| *MED13L* | ENST00000281928.3 | Up | 1.78 | chr12 | 116446685 | 116446746 | cds | 3.89E-02 |
| *SYBU* | ENST00000422135.1 | Up | 1.78 | chr8 | 110587640 | 110588209 | cds | 3.72E-02 |
| *NAA38* | ENST00000249299.2 | Up | 1.78 | chr7 | 117832047 | 117832226 | cds, utr3 | 8.71E-03 |
| *REPIN1* | ENST00000479668.1 | Up | 1.77 | chr7 | 150070830 | 150071128 | utr3 | 1.82E-04 |
| *ANKRD49* | ENST00000544612.1 | Up | 1.77 | chr11 | 94232629 | 94232749 | utr3 | 1.35E-02 |
| *DUSP7* | ENST00000495880.1 | Up | 1.77 | chr3 | 52083709 | 52083889 | utr3 | 1.41E-02 |
| *REPIN1* | ENST00000397281.2 | Up | 1.77 | chr7 | 150070833 | 150071133 | utr3 | 2.29E-04 |
| *REPIN1* | ENST00000425389.2 | Up | 1.77 | chr7 | 150070828 | 150071128 | utr3 | 2.00E-04 |
| *FRG1B* | ENST00000439954.2 | Up | 1.77 | chr20 | 29625899 | 29628413 | cds | 2.09E-02 |
| *PLEKHA4* | ENST00000355496.5 | Up | 1.76 | chr19 | 49368794 | 49371711 | utr5, cds | 5.50E-03 |
| *MGRN1* | ENST00000399577.5 | Up | 1.76 | chr16 | 4739807 | 4740107 | utr3 | 8.32E-03 |
| *MGRN1* | ENST00000415496.1 | Up | 1.76 | chr16 | 4739807 | 4740107 | utr3 | 8.32E-03 |
| *CDC40* | ENST00000368930.1 | Up | 1.76 | chr6 | 110550150 | 110575238 | utr3, cds | 3.55E-02 |
| *REPIN1* | ENST00000444957.1 | Up | 1.75 | chr7 | 150070832 | 150071133 | utr3 | 2.88E-04 |
| *C9orf114* | ENST00000467582.1 | Up | 1.75 | chr9 | 131584818 | 131584998 | utr3 | 1.95E-03 |
| *DST* | ENST00000370769.4 | Up | 1.75 | chr6 | 56492013 | 56492935 | cds | 3.72E-02 |
| *DST* | ENST00000421834.2 | Up | 1.75 | chr6 | 56492012 | 56492933 | cds | 3.72E-02 |
| *MGRN1* | ENST00000262370.7 | Up | 1.74 | chr16 | 4739809 | 4740108 | utr3 | 9.33E-03 |
| *AGTPBP1* | ENST00000337006.4 | Up | 1.74 | chr9 | 88247770 | 88248131 | utr3 | 4.90E-02 |
| *ZNF566* | ENST00000454319.1 | Up | 1.74 | chr19 | 36940078 | 36940766 | cds | 3.47E-02 |
| *STRADB* | ENST00000415688.1 | Up | 1.74 | chr2 | 202344776 | 202344924 | cds, utr3 | 1.38E-02 |
| *XPOT* | ENST00000332707.5 | Up | 1.73 | chr12 | 64843887 | 64844218 | utr3 | 3.72E-02 |
| *NDUFAF5* | ENST00000463598.1 | Up | 1.73 | chr20 | 13797567 | 13797874 | utr3, cds | 2.95E-02 |
| *SYBU* | ENST00000533171.1 | Up | 1.73 | chr8 | 110587664 | 110588200 | cds | 4.68E-02 |
| *DST* | ENST00000370754.5 | Up | 1.73 | chr6 | 56492003 | 56492925 | cds | 4.37E-02 |
| *SYBU* | ENST00000533895.1 | Up | 1.73 | chr8 | 110587664 | 110588200 | cds | 4.68E-02 |
| *SYBU* | ENST00000399066.3 | Up | 1.73 | chr8 | 110587664 | 110588203 | cds | 4.68E-02 |
| *RUSC1* | ENST00000368347.4 | Up | 1.73 | chr1 | 155300398 | 155300548 | utr3 | 8.71E-03 |
| *FEM1B* | ENST00000306917.4 | Up | 1.73 | chr15 | 68586945 | 68587066 | utr3 | 3.47E-02 |
| *C1orf35* | ENST00000272139.4 | Up | 1.73 | chr1 | 228288490 | 228288581 | utr3 | 3.98E-02 |
| *SH3BP2* | ENST00000356331.5 | Up | 1.73 | chr4 | 2838638 | 2838788 | utr3 | 4.47E-02 |
| *ARL16* | ENST00000570561.1 | Up | 1.72 | chr17 | 79648524 | 79648643 | utr3 | 1.70E-02 |
| *RUSC1* | ENST00000368352.5 | Up | 1.72 | chr1 | 155300397 | 155300547 | utr3 | 9.33E-03 |
| *ANKRD49* | ENST00000302755.4 | Up | 1.72 | chr11 | 94232638 | 94232728 | utr3 | 3.16E-02 |
| *MITD1* | ENST00000409107.1 | Up | 1.72 | chr2 | 99788028 | 99790469 | cds | 4.27E-03 |
| *BCAR1* | ENST00000568864.1 | Up | 1.71 | chr16 | 75276946 | 75298285 | utr5 | 2.19E-02 |
| *C9orf114* | ENST00000361256.5 | Up | 1.71 | chr9 | 131584806 | 131584986 | utr3 | 2.34E-03 |
| *JMJD1C* | ENST00000542921.1 | Up | 1.71 | chr10 | 64948977 | 64949157 | cds | 1.00E-02 |
| *JMJD1C* | ENST00000402544.1 | Up | 1.71 | chr10 | 64948977 | 64949157 | cds | 1.00E-02 |
| *RUSC1* | ENST00000368349.4 | Up | 1.71 | chr1 | 155300395 | 155300545 | utr3 | 1.05E-02 |
| *AP1M1* | ENST00000444449.2 | Up | 1.71 | chr19 | 16345977 | 16346156 | utr3 | 1.32E-02 |
| *ZNF566* | ENST00000434377.2 | Up | 1.71 | chr19 | 36940081 | 36940769 | cds | 4.17E-02 |
| *SLIT2* | ENST00000504154.1 | Up | 1.70 | chr4 | 20621909 | 20622150 | utr3 | 2.04E-02 |
| *EPC2* | ENST00000457184.1 | Up | 1.70 | chr2 | 149402675 | 149447835 | cds | 4.79E-02 |
| *C8orf59* | ENST00000421308.2 | Up | 1.70 | chr8 | 86126721 | 86127185 | cds, utr3 | 1.07E-03 |
| *FAM53C* | ENST00000239906.5 | Up | 1.69 | chr5 | 137680668 | 137681088 | cds | 3.98E-04 |
| *ZNF484* | ENST00000332591.6 | Up | 1.69 | chr9 | 95609275 | 95609813 | cds | 3.39E-02 |
| *FAM53C* | ENST00000434981.2 | Up | 1.69 | chr5 | 137680652 | 137681096 | cds | 2.63E-04 |
| *JMJD1C* | ENST00000399251.1 | Up | 1.69 | chr10 | 64948976 | 64949156 | utr3 | 1.23E-02 |
| *C8orf59* | ENST00000518091.1 | Up | 1.69 | chr8 | 86126737 | 86127203 | cds, utr3 | 1.07E-03 |
| *C8orf59* | ENST00000417663.2 | Up | 1.68 | chr8 | 86126721 | 86127188 | cds, utr3 | 1.07E-03 |
| *UTP15* | ENST00000543251.1 | Up | 1.68 | chr5 | 72875831 | 72876339 | cds, utr3 | 4.17E-03 |
| *ARL16* | ENST00000574938.1 | Up | 1.68 | chr17 | 79648427 | 79648659 | utr3 | 3.24E-03 |
| *UTP15* | ENST00000509005.1 | Up | 1.68 | chr5 | 72875831 | 72876339 | cds, utr3 | 4.17E-03 |
| *ZNF484* | ENST00000395505.2 | Up | 1.68 | chr9 | 95609283 | 95609823 | cds | 3.55E-02 |
| *AP1M1* | ENST00000291439.3 | Up | 1.68 | chr19 | 16345979 | 16346160 | utr3 | 1.62E-02 |
| *ZNF484* | ENST00000375495.3 | Up | 1.68 | chr9 | 95609275 | 95609814 | cds | 3.72E-02 |
| *ZNF484* | ENST00000395506.3 | Up | 1.68 | chr9 | 95609282 | 95609822 | cds | 3.63E-02 |
| *MITD1* | ENST00000289359.2 | Up | 1.67 | chr2 | 99788028 | 99790472 | cds | 8.32E-03 |
| *C8orf59* | ENST00000524353.1 | Up | 1.67 | chr8 | 86126736 | 86127201 | cds, utr3 | 1.20E-03 |
| *TRAPPC11* | ENST00000334690.6 | Up | 1.67 | chr4 | 184634594 | 184634745 | utr3 | 2.04E-02 |
| *SLC31A1* | ENST00000374212.4 | Up | 1.67 | chr9 | 116022893 | 116023073 | utr3 | 3.55E-02 |
| *SKIDA1* | ENST00000444772.3 | Up | 1.67 | chr10 | 21804184 | 21805023 | cds | 3.47E-02 |
| *TCF7L2* | ENST00000369397.4 | Up | 1.67 | chr10 | 114710240 | 114710510 | utr5 | 3.16E-02 |
| *MIOS* | ENST00000340080.4 | Up | 1.66 | chr7 | 7612459 | 7612638 | cds | 4.79E-02 |
| *PPP1R2* | ENST00000438848.1 | Up | 1.66 | chr3 | 195243722 | 195256613 | cds | 2.82E-04 |
| *MSH6* | ENST00000540021.1 | Up | 1.66 | chr2 | 48025820 | 48026180 | cds | 3.89E-04 |
| *JMJD1C* | ENST00000327520.7 | Up | 1.66 | chr10 | 64948963 | 64949173 | cds | 8.51E-03 |
| *JMJD1C* | ENST00000399262.2 | Up | 1.66 | chr10 | 64948969 | 64949180 | cds | 8.51E-03 |
| *AAMDC* | ENST00000525034.1 | Up | 1.66 | chr11 | 77583227 | 77583398 | utr3, cds | 3.72E-02 |
| *RUSC1* | ENST00000292254.4 | Up | 1.65 | chr1 | 155300401 | 155300550 | utr3 | 1.45E-02 |
| *TRAPPC11* | ENST00000357207.4 | Up | 1.65 | chr4 | 184634593 | 184634744 | utr3 | 2.24E-02 |
| *ZC3H6* | ENST00000343936.4 | Up | 1.65 | chr2 | 113069422 | 113079342 | cds | 3.02E-02 |
| *EXOSC8* | ENST00000481013.1 | Up | 1.64 | chr13 | 37583458 | 37583576 | utr3 | 4.47E-02 |
| *UTP15* | ENST00000296792.4 | Up | 1.64 | chr5 | 72875815 | 72876356 | cds, utr3 | 4.79E-03 |
| *MITD1* | ENST00000422537.2 | Up | 1.64 | chr2 | 99788013 | 99797302 | cds | 7.76E-03 |
| *MAP3K10* | ENST00000253055.3 | Up | 1.64 | chr19 | 40720884 | 40721452 | utr3, cds | 9.33E-03 |
| *ARAP1* | ENST00000429686.1 | Up | 1.64 | chr11 | 72425216 | 72425366 | utr5 | 1.86E-02 |
| *LDB1* | ENST00000361198.5 | Up | 1.63 | chr10 | 103874287 | 103874555 | utr5 | 2.51E-02 |
| *PYCR2* | ENST00000343818.6 | Up | 1.63 | chr1 | 226107577 | 226107963 | utr3 | 8.13E-05 |
| *ZNF784* | ENST00000325351.4 | Up | 1.62 | chr19 | 56132850 | 56133296 | cds, utr3 | 1.38E-02 |
| *AAMDC* | ENST00000526415.1 | Up | 1.62 | chr11 | 77583224 | 77583398 | utr3, cds | 4.37E-02 |
| *MLST8* | ENST00000301724.10 | Up | 1.62 | chr16 | 2258814 | 2258994 | utr3 | 2.00E-02 |
| *ARAP1* | ENST00000426523.1 | Up | 1.61 | chr11 | 72425215 | 72425366 | utr5 | 2.14E-02 |
| *ARAP1* | ENST00000334211.8 | Up | 1.61 | chr11 | 72425215 | 72425366 | utr5 | 2.14E-02 |
| *AAMDC* | ENST00000393427.2 | Up | 1.61 | chr11 | 77583229 | 77583407 | utr3, cds | 4.79E-02 |
| *UBAP2* | ENST00000412543.1 | Up | 1.61 | chr9 | 33963762 | 33996262 | cds | 1.58E-03 |
| *TMEM170A* | ENST00000561878.1 | Up | 1.61 | chr16 | 75481095 | 75485581 | utr3, cds | 1.12E-03 |
| *ZNF784* | ENST00000591479.1 | Up | 1.60 | chr19 | 56132841 | 56133291 | utr3 | 1.70E-02 |
| *UBA5* | ENST00000264991.4 | Up | 1.60 | chr3 | 132395291 | 132395382 | cds, utr3 | 3.09E-02 |
| *UBE4A* | ENST00000252108.3 | Up | 1.59 | chr11 | 118267053 | 118267383 | utr3, cds | 3.63E-02 |
| *RAB5A* | ENST00000443878.1 | Up | 1.59 | chr3 | 19988799 | 19988973 | utr5 | 7.59E-03 |
| *MLST8* | ENST00000382450.4 | Up | 1.59 | chr16 | 2258865 | 2259013 | cds, utr3 | 1.74E-02 |
| *MLST8* | ENST00000564088.1 | Up | 1.59 | chr16 | 2258865 | 2259013 | cds, utr3 | 1.74E-02 |
| *UBE4A* | ENST00000431736.2 | Up | 1.59 | chr11 | 118267055 | 118267385 | utr3, cds | 3.80E-02 |
| *METTL18* | ENST00000454472.1 | Up | 1.59 | chr1 | 169762282 | 169762693 | cds | 4.27E-02 |
| *UBAP2* | ENST00000418786.2 | Up | 1.59 | chr9 | 33963773 | 33996272 | cds | 2.24E-03 |
| *CENPJ* | ENST00000418179.1 | Up | 1.59 | chr13 | 25463508 | 25473676 | cds | 3.80E-02 |
| *UBAP2* | ENST00000449054.1 | Up | 1.58 | chr9 | 33971708 | 33996250 | cds | 1.32E-03 |
| *UBAP2* | ENST00000379239.4 | Up | 1.58 | chr9 | 33963765 | 33996263 | utr5 | 2.24E-03 |
| *ZC3H6* | ENST00000409871.1 | Up | 1.58 | chr2 | 113069415 | 113079335 | cds | 4.07E-02 |
| *STRADB* | ENST00000194530.3 | Up | 1.58 | chr2 | 202343318 | 202344944 | utr3, cds | 1.07E-02 |
| *TMEM170A* | ENST00000567796.1 | Up | 1.58 | chr16 | 75481099 | 75485578 | cds, utr3 | 2.09E-03 |
| *NOD1* | ENST00000222823.4 | Up | 1.58 | chr7 | 30464142 | 30464413 | utr3 | 2.95E-02 |
| *TMEM170A* | ENST00000569540.1 | Up | 1.58 | chr16 | 75481105 | 75485593 | utr3, cds | 1.48E-03 |
| *MLST8* | ENST00000565250.1 | Up | 1.57 | chr16 | 2258873 | 2258994 | cds, utr3 | 2.88E-02 |
| *RRNAD1* | ENST00000368218.4 | Up | 1.57 | chr1 | 156706563 | 156706741 | utr3 | 9.12E-03 |
| *MSH6* | ENST00000538136.1 | Up | 1.57 | chr2 | 48023101 | 48026160 | cds, utr5 | 1.38E-04 |
| *UBAP2* | ENST00000360802.1 | Up | 1.57 | chr9 | 33971690 | 33996262 | cds | 1.15E-03 |
| *BAP1* | ENST00000296288.5 | Up | 1.57 | chr3 | 52435041 | 52435161 | utr3 | 4.17E-02 |
| *SLC2A4RG* | ENST00000266077.2 | Up | 1.57 | chr20 | 62373821 | 62374232 | cds | 5.01E-03 |
| *MLST8* | ENST00000569417.1 | Up | 1.57 | chr16 | 2258878 | 2258998 | utr3 | 3.24E-02 |
| *PLEKHA4* | ENST00000263265.6 | Up | 1.57 | chr19 | 49368787 | 49371889 | utr5, cds | 1.26E-02 |
| *ZNF841* | ENST00000359973.2 | Up | 1.57 | chr19 | 52570314 | 52570786 | cds | 9.77E-04 |
| *UBAP2* | ENST00000379238.1 | Up | 1.56 | chr9 | 33971724 | 33996266 | cds | 1.58E-03 |
| *MLST8* | ENST00000301725.7 | Up | 1.56 | chr16 | 2258859 | 2259009 | utr3 | 2.14E-02 |
| *MSH6* | ENST00000234420.5 | Up | 1.56 | chr2 | 48023111 | 48026167 | cds | 2.04E-04 |
| *ECD* | ENST00000454759.2 | Up | 1.56 | chr10 | 74894281 | 74894461 | utr3, cds | 2.75E-02 |
| *RRNAD1* | ENST00000368216.4 | Up | 1.56 | chr1 | 156706562 | 156706741 | utr3 | 1.05E-02 |
| *RRNAD1* | ENST00000476229.1 | Up | 1.56 | chr1 | 156706562 | 156706741 | utr3 | 1.05E-02 |
| *C5orf51* | ENST00000381647.2 | Up | 1.56 | chr5 | 41919012 | 41919253 | utr3 | 2.88E-02 |
| *MLLT4* | ENST00000366806.2 | Up | 1.56 | chr6 | 168272906 | 168276044 | cds | 1.51E-03 |
| *ZNF605* | ENST00000392321.3 | Up | 1.56 | chr12 | 133501662 | 133502141 | utr3, cds | 2.19E-02 |
| *LRRC47* | ENST00000378251.1 | Up | 1.55 | chr1 | 3697229 | 3697676 | utr3, cds | 1.95E-03 |
| *AIMP1* | ENST00000394701.4 | Up | 1.55 | chr4 | 107268748 | 107268957 | utr3, cds | 3.72E-03 |
| *CSNK2A2* | ENST00000262506.3 | Up | 1.55 | chr16 | 58191810 | 58192079 | utr3 | 3.63E-03 |
| *MDM4* | ENST00000444261.1 | Up | 1.55 | chr1 | 204518287 | 204518516 | cds | 1.48E-02 |
| *CAPRIN2* | ENST00000537108.1 | Up | 1.55 | chr12 | 30878994 | 30881913 | cds | 3.24E-02 |
| *ZNF7* | ENST00000325241.6 | Up | 1.55 | chr8 | 146068364 | 146068605 | utr3, cds | 6.61E-03 |
| *AIMP1* | ENST00000442366.1 | Up | 1.55 | chr4 | 107268727 | 107268968 | utr3, cds | 2.75E-03 |
| *AIMP1* | ENST00000358008.3 | Up | 1.55 | chr4 | 107268724 | 107268963 | utr3, cds | 2.69E-03 |
| *KDM5A* | ENST00000399788.2 | Up | 1.55 | chr12 | 393401 | 393672 | utr3 | 1.35E-02 |
| *CAPRIN2* | ENST00000433722.2 | Up | 1.55 | chr12 | 30878968 | 30881915 | cds | 3.09E-02 |
| *ZNF829* | ENST00000520965.1 | Up | 1.54 | chr19 | 37380344 | 37381634 | utr3 | 1.07E-02 |
| *TMEM170A* | ENST00000357613.4 | Up | 1.54 | chr16 | 75481103 | 75485660 | utr3, cds | 2.24E-03 |
| *ZNF605* | ENST00000360187.4 | Up | 1.54 | chr12 | 133501663 | 133502142 | utr3, cds | 2.40E-02 |
| *ZDHHC12* | ENST00000372663.4 | Up | 1.54 | chr9 | 131483147 | 131483296 | utr3 | 1.70E-02 |
| *ECD* | ENST00000372979.4 | Up | 1.54 | chr10 | 74894307 | 74894486 | utr3, cds | 1.95E-02 |
| *ZDHHC12* | ENST00000372672.2 | Up | 1.54 | chr9 | 131483147 | 131483298 | utr3 | 1.70E-02 |
| *UBA5* | ENST00000356232.4 | Up | 1.54 | chr3 | 132394745 | 132395444 | utr3, cds | 1.15E-02 |
| *STRADB* | ENST00000392249.2 | Up | 1.54 | chr2 | 202343305 | 202344939 | utr3, cds | 1.41E-02 |
| *MLLT4* | ENST00000344191.4 | Up | 1.54 | chr6 | 168272907 | 168276045 | cds | 1.91E-03 |
| *B3GNT5* | ENST00000465010.1 | Up | 1.53 | chr3 | 182988379 | 182988620 | cds | 4.68E-02 |
| *VPS37B* | ENST00000371248.3 | Up | 1.53 | chr12 | 123351961 | 123353043 | cds | 1.91E-02 |
| *MLST8* | ENST00000397124.1 | Up | 1.53 | chr16 | 2258863 | 2259013 | cds, utr3 | 2.69E-02 |
| *SMG9* | ENST00000602222.1 | Up | 1.53 | chr19 | 44251659 | 44251901 | cds | 4.90E-02 |
| *ZNF7* | ENST00000544249.1 | Up | 1.53 | chr8 | 146068366 | 146068605 | cds, utr3 | 8.13E-03 |
| *EP400* | ENST00000330386.6 | Up | 1.53 | chr12 | 132446015 | 132466139 | cds | 6.92E-03 |
| *GGA3* | ENST00000578348.1 | Up | 1.53 | chr17 | 73233496 | 73233616 | utr3, cds | 3.16E-02 |
| *ZNF7* | ENST00000446747.2 | Up | 1.53 | chr8 | 146068365 | 146068605 | utr3, cds | 8.32E-03 |
| *TSC1* | ENST00000298552.3 | Up | 1.53 | chr9 | 135766734 | 135767155 | utr3 | 2.88E-03 |
| *PIBF1* | ENST00000326291.6 | Up | 1.52 | chr13 | 73396024 | 73467969 | cds | 4.68E-03 |
| *NDUFAF2* | ENST00000511107.1 | Up | 1.52 | chr5 | 60241179 | 60448824 | cds, utr3 | 1.45E-02 |
| *KIAA0391* | ENST00000321130.10 | Up | 1.52 | chr14 | 35593064 | 35596757 | cds | 3.98E-02 |
| *ARIH1* | ENST00000379887.4 | Up | 1.52 | chr15 | 72875950 | 72876011 | utr3 | 4.27E-02 |
| *WWC2* | ENST00000504005.1 | Up | 1.52 | chr4 | 184174982 | 184182522 | cds | 3.09E-03 |
| *MLLT4* | ENST00000392112.1 | Up | 1.52 | chr6 | 168272909 | 168276047 | cds | 2.40E-03 |
| *B3GNT5* | ENST00000326505.3 | Up | 1.51 | chr3 | 182988340 | 182988610 | cds | 3.89E-02 |
| *MLLT4* | ENST00000400824.4 | Up | 1.51 | chr6 | 168272926 | 168276058 | cds | 2.24E-03 |
| *NAGK* | ENST00000443872.2 | Up | 1.51 | chr2 | 71305626 | 71305715 | utr3, cds | 3.80E-03 |
| *MRPS35* | ENST00000538315.1 | Up | 1.51 | chr12 | 27908192 | 27908370 | utr3 | 1.91E-02 |
| *UBA5* | ENST00000494238.2 | Up | 1.51 | chr3 | 132394758 | 132395458 | cds, utr3 | 1.38E-02 |
| *PPP5C* | ENST00000391919.1 | Up | 1.51 | chr19 | 46893960 | 46894109 | utr3 | 4.68E-02 |
| *ZDHHC12* | ENST00000372667.5 | Up | 1.51 | chr9 | 131483148 | 131483297 | utr3 | 2.19E-02 |
| *C8orf59* | ENST00000431163.2 | Up | 1.51 | chr8 | 86126718 | 86129648 | cds, utr3 | 5.50E-03 |
| *KIAA0391* | ENST00000603544.1 | Up | 1.51 | chr14 | 35593068 | 35596733 | cds | 4.79E-02 |
| *PPP5C* | ENST00000012443.4 | Up | 1.51 | chr19 | 46893960 | 46894109 | utr3 | 4.68E-02 |
| *OFD1* | ENST00000380550.3 | Up | 1.51 | chrX | 13785279 | 13787390 | utr3, cds | 1.02E-04 |
| *PHC3* | ENST00000484931.1 | Up | 1.51 | chr3 | 169847095 | 169847305 | cds | 4.07E-02 |
| *RBMS2* | ENST00000552247.2 | Up | 1.51 | chr12 | 56983110 | 56983287 | utr3 | 2.69E-03 |
| *ZNF146* | ENST00000456324.1 | Up | 1.51 | chr19 | 36729402 | 36729673 | utr3 | 5.37E-03 |
| *PTPN2* | ENST00000587703.1 | Up | 1.51 | chr18 | 12785683 | 12802053 | utr3, cds | 2.45E-03 |
| *ZNF561* | ENST00000326044.5 | Up | 1.50 | chr19 | 9721617 | 9721974 | utr3 | 3.39E-02 |
| *ZNF561* | ENST00000354661.4 | Up | 1.50 | chr19 | 9721617 | 9721974 | cds, utr5 | 3.39E-02 |
| *ZNF146* | ENST00000443387.2 | Up | 1.50 | chr19 | 36729405 | 36729676 | utr3 | 5.62E-03 |
| *COPRS* | ENST00000378634.2 | Up | 1.50 | chr17 | 30178889 | 30179068 | utr3 | 3.80E-02 |
| *MASTL* | ENST00000342386.6 | Up | 1.50 | chr10 | 27459060 | 27459480 | cds | 3.24E-02 |
| *RBMS2* | ENST00000550726.1 | Up | 1.50 | chr12 | 56983108 | 56983287 | utr3 | 2.82E-03 |
| *RNF145* | ENST00000274542.2 | Up | 1.50 | chr5 | 158630586 | 158634834 | utr5, cds | 3.31E-02 |
| *OFD1* | ENST00000380567.1 | Up | 1.50 | chrX | 13785272 | 13787382 | utr3, cds | 1.10E-04 |
| *SLC6A2* | ENST00000568943.1 | Down | 0.07 | chr16 | 55689515 | 55689784 | utr5 | 1.41E-02 |
| *NPIPB6* | ENST00000533640.1 | Down | 0.08 | chr16 | 28353905 | 28354053 | utr3, cds | 7.59E-03 |
| *LEKR1* | ENST00000491763.1 | Down | 0.09 | chr3 | 156544207 | 156544506 | utr5 | 1.38E-02 |
| *MBLAC2* | ENST00000514906.1 | Down | 0.09 | chr5 | 89769658 | 89769897 | cds | 2.19E-02 |
| *TSSK6* | ENST00000360913.3 | Down | 0.10 | chr19 | 19626179 | 19626300 | cds, utr5 | 3.16E-02 |
| *LRRC3* | ENST00000291592.4 | Down | 0.10 | chr21 | 45877072 | 45877311 | cds, utr3 | 3.47E-02 |
| *RSPO1* | ENST00000401070.1 | Down | 0.12 | chr1 | 38077963 | 38078322 | utr3 | 4.90E-02 |
| *AC016586.1* | ENST00000535853.1 | Down | 0.13 | chr19 | 4041364 | 4041783 | utr3 | 6.17E-03 |
| *TEX40* | ENST00000328404.6 | Down | 0.14 | chr11 | 64071267 | 64072238 | cds, utr3 | 3.16E-02 |
| *RPP25* | ENST00000322177.5 | Down | 0.15 | chr15 | 75246876 | 75246966 | utr3 | 4.90E-02 |
| *CASP10* | ENST00000360132.3 | Down | 0.16 | chr2 | 202083176 | 202083327 | utr3 | 2.14E-02 |
| *IFNLR1* | ENST00000374421.3 | Down | 0.18 | chr1 | 24483619 | 24483797 | utr3, cds | 3.80E-02 |
| *PNPT1* | ENST00000447944.2 | Down | 0.18 | chr2 | 55861429 | 55862026 | utr3 | 1.86E-02 |
| *GPR17* | ENST00000544369.1 | Down | 0.18 | chr2 | 128408390 | 128408481 | cds | 3.98E-02 |
| *GPR17* | ENST00000272644.3 | Down | 0.18 | chr2 | 128408390 | 128408481 | cds | 3.98E-02 |
| *FKBP14* | ENST00000222803.5 | Down | 0.19 | chr7 | 30052801 | 30053131 | utr3 | 4.57E-02 |
| *IFNLR1* | ENST00000327575.2 | Down | 0.19 | chr1 | 24483617 | 24483796 | utr3 | 3.98E-02 |
| *IFNLR1* | ENST00000327535.1 | Down | 0.19 | chr1 | 24483489 | 24483789 | utr3, cds | 2.45E-02 |
| *C9orf85* | ENST00000486911.2 | Down | 0.19 | chr9 | 74598038 | 74598248 | utr3 | 4.37E-02 |
| *C6orf132* | ENST00000356542.5 | Down | 0.19 | chr6 | 42094114 | 42096509 | utr3, cds | 1.38E-02 |
| *EPB41* | ENST00000373798.1 | Down | 0.21 | chr1 | 29444726 | 29444966 | utr3 | 4.90E-02 |
| *ATAD5* | ENST00000321990.4 | Down | 0.22 | chr17 | 29192776 | 29196324 | cds | 1.78E-02 |
| *KIF1B* | ENST00000377093.4 | Down | 0.22 | chr1 | 10367428 | 10367788 | utr3 | 1.26E-02 |
| *TMEM2* | ENST00000377055.1 | Down | 0.24 | chr9 | 74314107 | 74314310 | utr3, cds | 2.40E-02 |
| *PUS10* | ENST00000398658.2 | Down | 0.24 | chr2 | 61237431 | 61237850 | utr3 | 3.80E-02 |
| *GINS1* | ENST00000429262.2 | Down | 0.24 | chr20 | 25388423 | 25397745 | utr5, cds | 3.02E-02 |
| *GINS1* | ENST00000262460.4 | Down | 0.24 | chr20 | 25388422 | 25397744 | utr5, cds | 3.02E-02 |
| *TGIF2* | ENST00000373872.4 | Down | 0.24 | chr20 | 35221217 | 35221487 | utr3 | 2.29E-02 |
| *TGIF2* | ENST00000373874.2 | Down | 0.24 | chr20 | 35221218 | 35221488 | utr3 | 2.29E-02 |
| *GALNT4* | ENST00000413530.1 | Down | 0.26 | chr12 | 89917322 | 89917442 | cds | 3.47E-02 |
| *ZDHHC14* | ENST00000359775.5 | Down | 0.26 | chr6 | 158097617 | 158097918 | utr3 | 4.57E-02 |
| *NDUFA1* | ENST00000371437.4 | Down | 0.26 | chrX | 119005449 | 119005597 | utr5 | 4.90E-02 |
| *NPIPB4* | ENST00000415645.2 | Down | 0.27 | chr16 | 21847631 | 21847840 | cds | 1.12E-03 |
| *MEGF9* | ENST00000426959.1 | Down | 0.27 | chr9 | 123364229 | 123364590 | utr3 | 3.09E-02 |
| *MEGF9* | ENST00000373930.3 | Down | 0.27 | chr9 | 123364229 | 123364590 | utr3 | 3.09E-02 |
| *PBRM1* | ENST00000420148.1 | Down | 0.28 | chr3 | 52716243 | 52719904 | utr5 | 3.31E-02 |
| *LRCH3* | ENST00000438796.2 | Down | 0.28 | chr3 | 197613001 | 197613272 | utr3 | 4.57E-02 |
| *POC1B-GALNT4* | ENST00000547474.1 | Down | 0.28 | chr12 | 89917320 | 89917440 | cds, utr3 | 4.79E-02 |
| *FOXK1* | ENST00000328914.4 | Down | 0.28 | chr7 | 4809095 | 4809455 | utr3 | 9.33E-03 |
| *NPIPB4* | ENST00000451409.1 | Down | 0.28 | chr16 | 21847630 | 21847918 | cds | 4.07E-03 |
| *SDCCAG8* | ENST00000476722.2 | Down | 0.29 | chr1 | 243468335 | 243468692 | utr3, cds | 2.75E-02 |
| *GDPD1* | ENST00000284116.4 | Down | 0.29 | chr17 | 57351603 | 57351873 | utr3 | 4.57E-03 |
| *KIAA1328* | ENST00000280020.5 | Down | 0.30 | chr18 | 34805106 | 34805287 | utr3 | 4.37E-02 |
| *SFMBT1* | ENST00000482396.1 | Down | 0.30 | chr3 | 53079862 | 53080039 | utr5 | 4.17E-02 |
| *SFMBT1* | ENST00000358080.2 | Down | 0.30 | chr3 | 53079860 | 53080040 | utr5 | 4.17E-02 |
| *ZNF641* | ENST00000550342.1 | Down | 0.31 | chr12 | 48741730 | 48743909 | cds, utr5 | 1.32E-02 |
| *ZNF84* | ENST00000451927.1 | Down | 0.31 | GL000223.1 | 46829 | 47130 | utr3 | 1.95E-02 |
| *TRAPPC2P1* | ENST00000596755.1 | Down | 0.32 | chr19 | 57875702 | 57876175 | utr5 | 2.14E-02 |
| *ZNF493* | ENST00000355504.4 | Down | 0.33 | chr19 | 21607041 | 21607340 | cds | 1.23E-02 |
| *CROCC* | ENST00000445545.2 | Down | 0.33 | chr1 | 17263252 | 17264228 | cds | 1.35E-03 |
| *CLN3* | ENST00000569430.1 | Down | 0.33 | chr16 | 28506706 | 28506855 | utr5 | 3.24E-02 |
| *OBSCN* | ENST00000366706.2 | Down | 0.33 | chr1 | 228465218 | 228466434 | cds | 4.57E-02 |
| *CROCC* | ENST00000375541.5 | Down | 0.35 | chr1 | 17263245 | 17264915 | cds | 1.70E-03 |
| *ZNF493* | ENST00000392288.2 | Down | 0.35 | chr19 | 21607056 | 21607356 | cds | 1.62E-02 |
| *SLC33A1* | ENST00000392845.3 | Down | 0.35 | chr3 | 155539231 | 155539771 | utr3 | 3.63E-02 |
| *CCNJL* | ENST00000393977.3 | Down | 0.35 | chr5 | 159679914 | 159680363 | utr3 | 4.57E-02 |
| *ZNF641* | ENST00000448928.3 | Down | 0.36 | chr12 | 48741719 | 48744991 | cds, utr5 | 2.24E-02 |
| *UTP23* | ENST00000309822.2 | Down | 0.37 | chr8 | 117785791 | 117786179 | utr3 | 4.68E-02 |
| *TBCEL* | ENST00000528512.1 | Down | 0.37 | chr11 | 120894850 | 120916416 | cds, utr5 | 6.31E-03 |
| *ZNF526* | ENST00000301215.3 | Down | 0.38 | chr19 | 42729760 | 42729910 | cds | 9.77E-03 |
| *ADRBK2* | ENST00000324198.6 | Down | 0.39 | chr22 | 26121667 | 26122117 | utr3 | 3.89E-02 |
| *LRIG2* | ENST00000361127.5 | Down | 0.39 | chr1 | 113669852 | 113670661 | utr3 | 4.57E-03 |
| *SOGA2* | ENST00000306285.7 | Down | 0.39 | chr18 | 8784193 | 8784672 | utr5 | 1.95E-02 |
| *ZNF641* | ENST00000544117.2 | Down | 0.39 | chr12 | 48741726 | 48743837 | cds | 2.75E-02 |
| *ZNF641* | ENST00000301042.3 | Down | 0.39 | chr12 | 48741728 | 48743838 | cds | 2.75E-02 |
| *SOGA2* | ENST00000359865.3 | Down | 0.39 | chr18 | 8784192 | 8784671 | cds | 1.95E-02 |
| *ZNF641* | ENST00000550181.1 | Down | 0.40 | chr12 | 48741725 | 48744993 | cds, utr5 | 3.02E-02 |
| *ZNF641* | ENST00000547026.1 | Down | 0.40 | chr12 | 48741734 | 48744584 | cds, utr5 | 3.02E-02 |
| *MEF2BNB* | ENST00000477565.3 | Down | 0.40 | chr19 | 19292928 | 19293108 | utr3 | 9.33E-03 |
| *CYP2U1* | ENST00000332884.6 | Down | 0.40 | chr4 | 108871586 | 108872097 | utr3 | 3.72E-03 |
| *UBE2V2* | ENST00000523111.2 | Down | 0.40 | chr8 | 48974931 | 48975232 | utr3 | 1.26E-02 |
| *LAMP2* | ENST00000371335.4 | Down | 0.40 | chrX | 119571631 | 119571990 | utr3 | 9.12E-04 |
| *ZNF641* | ENST00000548932.1 | Down | 0.41 | chr12 | 48741735 | 48743838 | cds | 3.24E-02 |
| *SLC24A1* | ENST00000505666.2 | Down | 0.41 | chr15 | 65947173 | 65947410 | utr3 | 3.98E-02 |
| *STAT3* | ENST00000585517.1 | Down | 0.42 | chr17 | 40466871 | 40467231 | utr3 | 5.01E-04 |
| *MDM2* | ENST00000462284.1 | Down | 0.42 | chr12 | 69238615 | 69238826 | utr3 | 4.79E-02 |
| *IRF1* | ENST00000245414.4 | Down | 0.42 | chr5 | 131817658 | 131818643 | utr3 | 3.47E-03 |
| *RAB3D* | ENST00000222120.3 | Down | 0.42 | chr19 | 11433528 | 11434186 | utr3 | 2.63E-03 |
| *ZNF629* | ENST00000262525.4 | Down | 0.42 | chr16 | 30794358 | 30794569 | cds | 3.55E-02 |
| *CTD-2368P22.1* | ENST00000547364.1 | Down | 0.42 | chr19 | 58520827 | 58521063 | utr3 | 4.90E-02 |
| *PLEKHG2* | ENST00000425673.1 | Down | 0.43 | chr19 | 39918604 | 39919054 | utr3 | 2.88E-02 |
| *CYP2U1* | ENST00000508453.1 | Down | 0.43 | chr4 | 108871602 | 108872109 | utr3 | 6.03E-03 |
| *ZNF354C* | ENST00000315475.6 | Down | 0.43 | chr5 | 178505695 | 178505995 | cds | 1.55E-02 |
| *ZYG11B* | ENST00000294353.6 | Down | 0.43 | chr1 | 53289566 | 53290496 | utr3 | 3.72E-04 |
| *TBCEL* | ENST00000422003.2 | Down | 0.44 | chr11 | 120894889 | 120916511 | cds, utr5 | 8.32E-03 |
| *ZNF260* | ENST00000592282.1 | Down | 0.45 | chr19 | 37001959 | 37002528 | utr3 | 3.72E-02 |
| *VMA21* | ENST00000330374.6 | Down | 0.46 | chrX | 150576402 | 150576761 | utr3 | 3.63E-03 |
| *TCF7* | ENST00000517855.1 | Down | 0.46 | chr5 | 133482161 | 133482368 | utr3, cds | 3.89E-02 |
| *CTBP2* | ENST00000494626.2 | Down | 0.46 | chr10 | 126822124 | 126849068 | utr5 | 3.09E-02 |
| *N4BP2* | ENST00000261435.6 | Down | 0.46 | chr4 | 40156693 | 40157174 | utr3 | 2.29E-02 |
| *WDR52* | ENST00000393845.2 | Down | 0.46 | chr3 | 113005986 | 113006287 | utr3 | 1.38E-02 |
| *MACF1* | ENST00000289893.4 | Down | 0.46 | chr1 | 39802968 | 39806289 | cds | 3.63E-02 |
| *STARD10* | ENST00000400925.2 | Down | 0.47 | chr11 | 72466373 | 72466743 | cds, utr3 | 6.61E-03 |
| *NINL* | ENST00000278886.6 | Down | 0.47 | chr20 | 25462630 | 25477395 | cds | 3.39E-02 |
| *NINL* | ENST00000422516.1 | Down | 0.47 | chr20 | 25462630 | 25477395 | cds | 3.39E-02 |
| *CEP250* | ENST00000342580.4 | Down | 0.47 | chr20 | 34061351 | 34064309 | cds | 1.12E-02 |
| *TCF7* | ENST00000342854.5 | Down | 0.48 | chr5 | 133482016 | 133482315 | utr3 | 9.77E-03 |
| *ARSD* | ENST00000381154.1 | Down | 0.48 | chrX | 2824859 | 2825040 | utr3 | 4.68E-02 |
| *MGAT4A* | ENST00000495056.2 | Down | 0.48 | chr2 | 99342698 | 99342936 | cds, utr5 | 2.75E-02 |
| *BOC* | ENST00000273395.4 | Down | 0.48 | chr3 | 113005998 | 113006298 | utr3 | 1.91E-02 |
| *BOC* | ENST00000495514.1 | Down | 0.48 | chr3 | 113005998 | 113006298 | utr3 | 1.91E-02 |
| *NCDN* | ENST00000373253.3 | Down | 0.48 | chr1 | 36032223 | 36032373 | utr3 | 1.91E-02 |
| *ZFC3H1* | ENST00000548100.1 | Down | 0.48 | chr12 | 72057308 | 72057545 | utr5, cds | 1.15E-03 |
| *NCDN* | ENST00000373243.2 | Down | 0.48 | chr1 | 36032223 | 36032374 | utr3 | 1.91E-02 |
| *TCF7* | ENST00000378560.4 | Down | 0.49 | chr5 | 133482037 | 133482336 | utr3 | 1.45E-02 |
| *IQCG* | ENST00000453254.1 | Down | 0.49 | chr3 | 197640712 | 197665427 | cds | 4.17E-02 |
| *KIAA1467* | ENST00000197268.8 | Down | 0.49 | chr12 | 13234859 | 13235368 | utr3 | 2.19E-03 |
| *BCL7A* | ENST00000261822.4 | Down | 0.49 | chr12 | 122497179 | 122497773 | utr3 | 1.58E-02 |
| *TCF7* | ENST00000520958.1 | Down | 0.49 | chr5 | 133482024 | 133482264 | utr3 | 2.88E-02 |
| *CEP250* | ENST00000425934.1 | Down | 0.49 | chr20 | 34061356 | 34064314 | cds | 1.51E-02 |
| *YTHDF2* | ENST00000496288.1 | Down | 0.50 | chr1 | 29063535 | 29063653 | utr5, cds | 4.47E-02 |
| *PMM1* | ENST00000216259.7 | Down | 0.50 | chr22 | 41973133 | 41973252 | utr3 | 9.77E-03 |
| *WDR44* | ENST00000254029.3 | Down | 0.50 | chrX | 117527055 | 117528048 | cds | 3.09E-02 |
| *CEP250* | ENST00000397527.1 | Down | 0.50 | chr20 | 34061368 | 34064297 | cds | 2.04E-02 |
| *BCL7A* | ENST00000538010.1 | Down | 0.50 | chr12 | 122497169 | 122497767 | utr3 | 2.04E-02 |
| *ZFC3H1* | ENST00000378743.3 | Down | 0.50 | chr12 | 72057298 | 72057569 | utr5, cds | 1.23E-03 |
| *TCF7* | ENST00000395023.1 | Down | 0.50 | chr5 | 133482033 | 133482333 | utr3 | 1.74E-02 |
| *NCDN* | ENST00000356090.4 | Down | 0.50 | chr1 | 36032230 | 36032380 | utr3 | 2.63E-02 |
| *TRIM2* | ENST00000338700.5 | Down | 0.50 | chr4 | 154216720 | 154217170 | cds | 3.72E-02 |
| *SYNJ2BP* | ENST00000256366.4 | Down | 0.51 | chr14 | 70838147 | 70838504 | utr3 | 2.95E-02 |
| *ASB13* | ENST00000357700.6 | Down | 0.51 | chr10 | 5681999 | 5682450 | utr3 | 9.12E-03 |
| *PUS7* | ENST00000356362.2 | Down | 0.51 | chr7 | 105097317 | 105097618 | utr3 | 4.17E-02 |
| *THAP2* | ENST00000308086.2 | Down | 0.51 | chr12 | 72057297 | 72057537 | utr5 | 2.24E-03 |
| *RP11-166B2.1* | ENST00000399147.4 | Down | 0.51 | chr16 | 12021229 | 12021558 | utr3, cds | 1.55E-02 |
| *NIP7* | ENST00000254940.5 | Down | 0.51 | chr16 | 69376183 | 69376629 | utr3 | 1.00E-02 |
| *EEF2K* | ENST00000263026.5 | Down | 0.52 | chr16 | 22295653 | 22296072 | utr3 | 3.47E-02 |
| *AC007390.5* | ENST00000392061.2 | Down | 0.52 | chr2 | 37431505 | 37431882 | utr3 | 2.14E-03 |
| *CTSB* | ENST00000353047.6 | Down | 0.52 | chr8 | 11700032 | 11700748 | utr3 | 1.10E-10 |
| *CYB5D2* | ENST00000301391.3 | Down | 0.52 | chr17 | 4046579 | 4047087 | cds, utr5 | 1.48E-02 |
| *CTSB* | ENST00000434271.1 | Down | 0.52 | chr8 | 11700032 | 11700751 | utr3 | 1.00E-10 |
| *CSPG4* | ENST00000308508.5 | Down | 0.52 | chr15 | 75966901 | 75967202 | utr3 | 1.82E-05 |
| *CFLAR* | ENST00000341582.6 | Down | 0.52 | chr2 | 201980959 | 201981138 | utr5 | 3.31E-03 |
| *BTBD7* | ENST00000298896.3 | Down | 0.52 | chr14 | 93762477 | 93799438 | utr5 | 1.55E-02 |
| *WDR44* | ENST00000371822.5 | Down | 0.52 | chrX | 117527066 | 117528058 | cds | 4.37E-02 |
| *SH3BP1* | ENST00000357436.4 | Down | 0.52 | chr22 | 38035540 | 38035779 | utr5 | 4.17E-02 |
| *PARP14* | ENST00000494811.1 | Down | 0.52 | chr3 | 122399792 | 122404110 | cds | 8.32E-03 |
| *TRIM2* | ENST00000437508.2 | Down | 0.52 | chr4 | 154216695 | 154236683 | cds | 3.16E-02 |
| *HEBP2* | ENST00000607197.1 | Down | 0.53 | chr6 | 138737367 | 138737818 | utr3 | 3.39E-02 |
| *DSN1* | ENST00000373750.4 | Down | 0.53 | chr20 | 35399560 | 35402158 | utr5, cds | 3.98E-02 |
| *FLJ00104* | ENST00000446344.1 | Down | 0.53 | chr16 | 87735282 | 87735582 | cds | 3.24E-02 |
| *TADA2B* | ENST00000310074.7 | Down | 0.53 | chr4 | 7059258 | 7059679 | utr3 | 1.29E-02 |
| *ZFC3H1* | ENST00000552037.1 | Down | 0.53 | chr12 | 72057281 | 72057549 | utr5, cds | 2.51E-03 |
| *PARP14* | ENST00000474629.2 | Down | 0.53 | chr3 | 122399794 | 122404119 | cds | 8.51E-03 |
| *ZSWIM3* | ENST00000454862.2 | Down | 0.53 | chr20 | 44506616 | 44507212 | cds | 4.79E-02 |
| *TBX3* | ENST00000349155.2 | Down | 0.53 | chr12 | 115121908 | 115121969 | utr5 | 1.55E-02 |
| *TTLL3* | ENST00000383827.1 | Down | 0.53 | chr3 | 9860115 | 9860474 | utr5 | 2.75E-02 |
| *TCF7* | ENST00000395029.1 | Down | 0.53 | chr5 | 133482041 | 133482341 | utr3, cds | 2.75E-02 |
| *ZNF207* | ENST00000394670.4 | Down | 0.53 | chr17 | 30700966 | 30701685 | utr3 | 2.19E-02 |
| *CFLAR* | ENST00000309955.3 | Down | 0.54 | chr2 | 201980916 | 201981126 | utr5 | 1.17E-03 |
| *NIP7* | ENST00000254941.6 | Down | 0.54 | chr16 | 69376198 | 69376618 | utr3 | 2.00E-02 |
| *KBTBD8* | ENST00000417314.2 | Down | 0.54 | chr3 | 67054332 | 67058755 | cds | 2.63E-02 |
| *CNEP1R1* | ENST00000458059.3 | Down | 0.54 | chr16 | 50070040 | 50070400 | utr3 | 2.88E-02 |
| *TBCD* | ENST00000539345.2 | Down | 0.54 | chr17 | 80765525 | 80772772 | cds | 3.31E-02 |
| *ZNF785* | ENST00000470110.1 | Down | 0.55 | chr16 | 30590590 | 30593936 | cds, utr3 | 1.74E-02 |
| *SPPL3* | ENST00000353487.2 | Down | 0.55 | chr12 | 121200312 | 121200820 | utr3 | 4.37E-02 |
| *SPTY2D1* | ENST00000336349.5 | Down | 0.55 | chr11 | 18628337 | 18628697 | utr3 | 2.82E-02 |
| *PURB* | ENST00000395699.2 | Down | 0.55 | chr7 | 44918498 | 44918947 | utr3 | 7.94E-03 |
| *COBL* | ENST00000445054.1 | Down | 0.55 | chr7 | 51092882 | 51095691 | cds | 3.80E-03 |
| *MUM1* | ENST00000591806.1 | Down | 0.55 | chr19 | 1377175 | 1377445 | utr3 | 3.72E-02 |
| *ZNF628* | ENST00000391718.2 | Down | 0.55 | chr19 | 55992961 | 55993767 | cds | 2.88E-02 |
| *ZNF628* | ENST00000598519.1 | Down | 0.55 | chr19 | 55992961 | 55993767 | cds | 2.88E-02 |
| *DNAH1* | ENST00000420323.2 | Down | 0.55 | chr3 | 52425591 | 52426661 | cds | 2.63E-02 |
| *UGDH* | ENST00000316423.6 | Down | 0.56 | chr4 | 39501267 | 39501744 | utr3 | 1.91E-02 |
| *SPTB* | ENST00000389722.3 | Down | 0.56 | chr14 | 65213480 | 65213691 | utr3 | 4.27E-02 |
| *MPRIP* | ENST00000395811.5 | Down | 0.56 | chr17 | 17092548 | 17092938 | utr3 | 4.79E-02 |
| *KBTBD8* | ENST00000295568.4 | Down | 0.56 | chr3 | 67054352 | 67058741 | cds | 3.55E-02 |
| *SNX7* | ENST00000529992.1 | Down | 0.56 | chr1 | 99167457 | 99203941 | cds | 2.24E-02 |
| *FBF1* | ENST00000586717.1 | Down | 0.56 | chr17 | 73910054 | 73913882 | cds | 1.45E-02 |
| *C17orf70* | ENST00000425898.2 | Down | 0.56 | chr17 | 79514055 | 79514296 | cds | 2.19E-02 |
| *UGDH* | ENST00000506179.1 | Down | 0.56 | chr4 | 39501274 | 39501754 | utr3 | 1.95E-02 |
| *CNEP1R1* | ENST00000427478.2 | Down | 0.56 | chr16 | 50070036 | 50070394 | utr3 | 3.63E-02 |
| *C17orf70* | ENST00000327787.8 | Down | 0.56 | chr17 | 79514055 | 79514296 | cds | 2.19E-02 |
| *C17orf70* | ENST00000537152.1 | Down | 0.56 | chr17 | 79514054 | 79514295 | cds | 2.19E-02 |
| *ZNF629* | ENST00000262525.4 | Down | 0.56 | chr16 | 30793550 | 30793940 | cds | 4.37E-02 |
| *MUM1* | ENST00000311401.5 | Down | 0.56 | chr19 | 1377169 | 1377440 | utr3 | 4.47E-02 |
| *FLAD1* | ENST00000368431.3 | Down | 0.56 | chr1 | 154960128 | 154960661 | cds, utr5 | 1.58E-02 |
| *MED11* | ENST00000573708.1 | Down | 0.56 | chr17 | 4635193 | 4635372 | cds, utr3 | 4.47E-02 |
| *CAV2* | ENST00000222693.4 | Down | 0.56 | chr7 | 116147405 | 116147911 | utr3 | 2.69E-04 |
| *TBCD* | ENST00000397466.2 | Down | 0.57 | chr17 | 80765537 | 80772783 | cds, utr5 | 4.37E-02 |
| *MUM1* | ENST00000344663.3 | Down | 0.57 | chr19 | 1377175 | 1377445 | utr3 | 4.57E-02 |
| *MTFMT* | ENST00000220058.4 | Down | 0.57 | chr15 | 65294874 | 65295444 | utr3, cds | 4.68E-02 |
| *SNX7* | ENST00000306121.3 | Down | 0.57 | chr1 | 99203793 | 99203944 | cds | 2.57E-02 |
| *SSH1* | ENST00000326495.5 | Down | 0.57 | chr12 | 109178775 | 109179226 | utr3 | 2.82E-02 |
| *MTHFSD* | ENST00000543303.2 | Down | 0.57 | chr16 | 86565606 | 86565902 | utr3, cds | 4.27E-02 |
| *UBFD1* | ENST00000219638.4 | Down | 0.57 | chr16 | 23569315 | 23569583 | cds | 1.48E-03 |
| *SSH1* | ENST00000360239.3 | Down | 0.57 | chr12 | 109178774 | 109179225 | utr3 | 3.02E-02 |
| *TERF2* | ENST00000566051.1 | Down | 0.57 | chr16 | 69390236 | 69395374 | cds, utr3 | 1.02E-03 |
| *THADA* | ENST00000405006.4 | Down | 0.57 | chr2 | 43457990 | 43458170 | utr3, cds | 4.17E-02 |
| *UBFD1* | ENST00000395878.3 | Down | 0.58 | chr16 | 23569312 | 23569582 | cds | 1.58E-03 |
| *THADA* | ENST00000405975.2 | Down | 0.58 | chr2 | 43457974 | 43458185 | utr3, cds | 3.39E-02 |
| *NFATC2IP* | ENST00000320805.4 | Down | 0.58 | chr16 | 28977730 | 28978328 | utr3 | 3.24E-03 |
| *C9orf47* | ENST00000334490.5 | Down | 0.58 | chr9 | 91606550 | 91606887 | cds | 1.58E-02 |
| *CNEP1R1* | ENST00000565556.1 | Down | 0.58 | chr16 | 50070031 | 50070391 | utr3 | 4.57E-02 |
| *ROCK2* | ENST00000315872.6 | Down | 0.58 | chr2 | 11321652 | 11322042 | utr3 | 4.07E-03 |
| *POMGNT2* | ENST00000344697.2 | Down | 0.58 | chr3 | 43120785 | 43122072 | utr3, cds | 6.31E-04 |
| *VAMP3* | ENST00000470357.1 | Down | 0.58 | chr1 | 7832796 | 7833546 | utr5 | 8.91E-04 |
| *SUV420H1* | ENST00000458496.1 | Down | 0.58 | chr11 | 67953333 | 67981145 | cds, utr5 | 1.10E-02 |
| *C11orf54* | ENST00000528288.1 | Down | 0.58 | chr11 | 93494926 | 93495137 | utr3 | 3.31E-02 |
| *YME1L1* | ENST00000376016.3 | Down | 0.58 | chr10 | 27399531 | 27400429 | utr3 | 8.91E-07 |
| *POMGNT2* | ENST00000441964.1 | Down | 0.58 | chr3 | 43120783 | 43122063 | utr3, cds | 6.92E-04 |
| *SMO* | ENST00000249373.3 | Down | 0.58 | chr7 | 128852189 | 128852639 | utr3, cds | 4.90E-02 |
| *UBFD1* | ENST00000567264.1 | Down | 0.58 | chr16 | 23569287 | 23569580 | cds | 1.82E-03 |
| *SUGP2* | ENST00000452918.2 | Down | 0.59 | chr19 | 19103157 | 19103516 | utr3 | 2.88E-02 |
| *C9orf47* | ENST00000375851.2 | Down | 0.59 | chr9 | 91606569 | 91606876 | cds | 2.45E-02 |
| *CFLAR* | ENST00000340870.5 | Down | 0.59 | chr2 | 201980919 | 201981127 | utr5 | 5.89E-03 |
| *CFLAR* | ENST00000355558.4 | Down | 0.59 | chr2 | 201980919 | 201981127 | utr5 | 5.89E-03 |
| *TMEM186* | ENST00000333050.6 | Down | 0.59 | chr16 | 8889597 | 8890130 | cds, utr3 | 1.95E-04 |
| *CFLAR* | ENST00000443227.1 | Down | 0.59 | chr2 | 201980936 | 201981147 | utr5 | 3.47E-03 |
| *FBF1* | ENST00000319129.5 | Down | 0.59 | chr17 | 73910088 | 73913882 | cds | 2.63E-02 |
| *ARMCX5* | ENST00000536530.1 | Down | 0.59 | chrX | 101856945 | 101857747 | cds, utr5 | 6.31E-03 |
| *TBCD* | ENST00000355528.4 | Down | 0.59 | chr17 | 80765521 | 80772797 | cds | 4.27E-02 |
| *YME1L1* | ENST00000326799.3 | Down | 0.59 | chr10 | 27399531 | 27400456 | utr3 | 9.77E-07 |
| *LIMS1* | ENST00000393310.1 | Down | 0.59 | chr2 | 109302506 | 109303373 | utr3 | 3.47E-03 |
| *MRPS10* | ENST00000053468.3 | Down | 0.59 | chr6 | 42174805 | 42175311 | utr3 | 6.61E-03 |
| *TMEM33* | ENST00000504986.1 | Down | 0.59 | chr4 | 41960703 | 41961153 | utr3 | 2.82E-02 |
| *FCHSD1* | ENST00000435817.2 | Down | 0.59 | chr5 | 141019198 | 141019379 | utr3 | 7.59E-03 |
| *RAD1* | ENST00000382038.2 | Down | 0.59 | chr5 | 34908178 | 34908657 | utr3 | 9.55E-03 |
| *RAD1* | ENST00000341754.4 | Down | 0.59 | chr5 | 34908276 | 34908662 | utr3 | 3.55E-02 |
| *PI4KB* | ENST00000368872.1 | Down | 0.59 | chr1 | 151264954 | 151265193 | utr3 | 6.31E-04 |
| *THADA* | ENST00000415080.2 | Down | 0.60 | chr2 | 43457975 | 43458186 | utr3, cds | 4.27E-02 |
| *CWC25* | ENST00000225428.5 | Down | 0.60 | chr17 | 36956896 | 36957435 | utr3 | 2.29E-02 |
| *ARMCX5* | ENST00000604957.1 | Down | 0.60 | chrX | 101856931 | 101857740 | cds, utr5 | 6.92E-03 |
| *ARMCX5* | ENST00000246174.2 | Down | 0.60 | chrX | 101856935 | 101857743 | cds, utr5 | 6.92E-03 |
| *ARMCX5* | ENST00000537008.1 | Down | 0.60 | chrX | 101856929 | 101857768 | cds, utr5 | 6.17E-03 |
| *ADAM10* | ENST00000561288.1 | Down | 0.60 | chr15 | 59041818 | 59041877 | utr5 | 4.27E-02 |
| *ZNF408* | ENST00000311764.2 | Down | 0.60 | chr11 | 46727193 | 46727462 | utr3, cds | 3.80E-02 |
| *USP54* | ENST00000394811.2 | Down | 0.60 | chr10 | 75257986 | 75258915 | utr3, cds | 1.82E-02 |
| *ARMCX5* | ENST00000541409.1 | Down | 0.60 | chrX | 101856929 | 101857767 | cds, utr5 | 7.08E-03 |
| *CFLAR* | ENST00000341222.6 | Down | 0.60 | chr2 | 201980919 | 201981129 | utr5 | 8.51E-03 |
| *NEK8* | ENST00000268766.6 | Down | 0.60 | chr17 | 27069249 | 27070473 | utr3 | 5.50E-03 |
| *FBF1* | ENST00000389570.4 | Down | 0.61 | chr17 | 73910088 | 73913883 | cds | 3.24E-02 |
| *WWP2* | ENST00000569174.1 | Down | 0.61 | chr16 | 69905776 | 69921973 | cds | 3.47E-02 |
| *NUP93* | ENST00000564887.1 | Down | 0.61 | chr16 | 56832399 | 56855490 | cds, utr5 | 2.82E-02 |
| *RGS3* | ENST00000467805.2 | Down | 0.61 | chr9 | 116345911 | 116356755 | cds | 3.72E-02 |
| *COBL* | ENST00000265136.7 | Down | 0.61 | chr7 | 51093032 | 51095700 | cds | 1.86E-02 |
| *COBL* | ENST00000395542.2 | Down | 0.61 | chr7 | 51093032 | 51095699 | cds | 1.86E-02 |
| *C11orf54* | ENST00000528099.1 | Down | 0.61 | chr11 | 93494907 | 93495115 | utr3 | 4.27E-02 |
| *NUP93* | ENST00000542526.1 | Down | 0.61 | chr16 | 56832416 | 56855505 | cds, utr5 | 2.63E-02 |
| *COBL* | ENST00000431948.1 | Down | 0.61 | chr7 | 51093031 | 51095699 | cds | 1.86E-02 |
| *NUP93* | ENST00000567641.1 | Down | 0.61 | chr16 | 56832411 | 56855489 | cds, utr5 | 2.75E-02 |
| *CASP2* | ENST00000310447.5 | Down | 0.61 | chr7 | 143003320 | 143003770 | utr3 | 3.63E-02 |
| *PRDM2* | ENST00000505823.1 | Down | 0.61 | chr1 | 14099580 | 14149675 | cds, utr5 | 1.41E-02 |
| *PLEKHJ1* | ENST00000587394.2 | Down | 0.61 | chr19 | 2234044 | 2235987 | cds | 2.51E-04 |
| *ARMCX5* | ENST00000372742.1 | Down | 0.61 | chrX | 101856910 | 101857743 | cds, utr5 | 8.91E-03 |
| *PRDM2* | ENST00000503842.1 | Down | 0.61 | chr1 | 14095618 | 14149671 | cds, utr5 | 1.32E-02 |
| *C11orf54* | ENST00000331239.4 | Down | 0.61 | chr11 | 93494913 | 93495122 | utr3 | 4.90E-02 |
| *SOGA2* | ENST00000517570.1 | Down | 0.62 | chr18 | 8824827 | 8825635 | cds | 3.02E-02 |
| *OSMR* | ENST00000274276.3 | Down | 0.62 | chr5 | 38934784 | 38935264 | utr3 | 7.41E-03 |
| *ZNF511* | ENST00000368554.4 | Down | 0.62 | chr10 | 135125315 | 135165540 | cds | 4.79E-02 |
| *LRRC57* | ENST00000563454.1 | Down | 0.62 | chr15 | 42834959 | 42835643 | utr3 | 3.72E-02 |
| *ZSWIM8* | ENST00000603187.1 | Down | 0.62 | chr10 | 75552399 | 75552609 | cds | 2.29E-03 |
| *JRKL* | ENST00000458427.1 | Down | 0.62 | chr11 | 96123217 | 96123518 | utr5 | 3.47E-02 |
| *EXO5* | ENST00000358527.2 | Down | 0.62 | chr1 | 40980377 | 40980703 | cds | 4.37E-02 |
| *UBFD1* | ENST00000567212.1 | Down | 0.62 | chr16 | 23569305 | 23569575 | cds | 6.76E-03 |
| *USP54* | ENST00000408019.1 | Down | 0.62 | chr10 | 75257985 | 75258913 | utr3, cds | 2.51E-02 |
| *USP54* | ENST00000428547.1 | Down | 0.62 | chr10 | 75257985 | 75258912 | utr3, cds | 2.51E-02 |
| *TLK1* | ENST00000431350.2 | Down | 0.62 | chr2 | 171913034 | 171974342 | cds | 1.12E-06 |
| *WWP2* | ENST00000448661.1 | Down | 0.62 | chr16 | 69905771 | 69921969 | cds | 4.57E-02 |
| *TLK1* | ENST00000442919.2 | Down | 0.63 | chr2 | 171913010 | 171974348 | cds | 7.41E-07 |
| *NAGK* | ENST00000418807.3 | Down | 0.63 | chr2 | 71295806 | 71298918 | utr5, cds | 5.50E-06 |
| *DNM1L* | ENST00000381000.4 | Down | 0.63 | chr12 | 32897286 | 32897885 | utr3 | 7.24E-04 |
| *THOC2* | ENST00000441692.1 | Down | 0.63 | chrX | 122736451 | 122736930 | utr3 | 4.57E-02 |
| *GSTZ1* | ENST00000553586.1 | Down | 0.63 | chr14 | 77797648 | 77797827 | utr3 | 7.41E-03 |
| *UBE2Z* | ENST00000514948.1 | Down | 0.63 | chr17 | 46998621 | 47000459 | cds, utr3 | 2.04E-04 |
| *TOR1AIP2* | ENST00000367612.3 | Down | 0.63 | chr1 | 179810867 | 179811318 | utr3 | 2.34E-03 |
| *MOGS* | ENST00000409065.1 | Down | 0.63 | chr2 | 74692241 | 74692512 | cds, utr5 | 2.69E-03 |
| *USP40* | ENST00000251722.6 | Down | 0.63 | chr2 | 234385878 | 234386059 | utr3, cds | 1.66E-02 |
| *DNM1L* | ENST00000452533.2 | Down | 0.63 | chr12 | 32897286 | 32897886 | utr3 | 8.13E-04 |
| *SUGP2* | ENST00000601879.1 | Down | 0.63 | chr19 | 19103175 | 19103536 | utr3 | 4.47E-02 |
| *HNRNPUL1* | ENST00000599614.1 | Down | 0.63 | chr19 | 41778052 | 41782081 | cds | 3.02E-03 |
| *WWP2* | ENST00000359154.2 | Down | 0.63 | chr16 | 69905777 | 69921975 | cds | 4.68E-02 |
| *TLK1* | ENST00000521943.1 | Down | 0.63 | chr2 | 171913020 | 171974357 | cds | 1.45E-06 |
| *SUGP2* | ENST00000601879.1 | Down | 0.63 | chr19 | 19136074 | 19136255 | cds | 3.55E-02 |
| *MOGS* | ENST00000535045.1 | Down | 0.63 | chr2 | 74692250 | 74692489 | cds, utr5 | 3.55E-03 |
| *WWP2* | ENST00000542271.1 | Down | 0.63 | chr16 | 69905766 | 69921963 | cds | 4.90E-02 |
| *SHC1* | ENST00000368445.5 | Down | 0.63 | chr1 | 154942558 | 154942738 | cds | 5.37E-04 |
| *TATDN2* | ENST00000287652.4 | Down | 0.63 | chr3 | 10302026 | 10311815 | cds | 3.55E-03 |
| *USP40* | ENST00000450966.1 | Down | 0.64 | chr2 | 234385867 | 234386077 | utr3, cds | 1.26E-02 |
| *ADAM10* | ENST00000558004.1 | Down | 0.64 | chr15 | 59041708 | 59041885 | utr5, cds | 2.29E-03 |
| *DYNC1H1* | ENST00000360184.4 | Down | 0.64 | chr14 | 102499648 | 102499799 | cds | 1.05E-03 |
| *MRPL52* | ENST00000397496.3 | Down | 0.64 | chr14 | 23299458 | 23304158 | cds, utr3 | 6.31E-05 |
| *ZSWIM8* | ENST00000604729.1 | Down | 0.64 | chr10 | 75552394 | 75552604 | cds | 3.09E-03 |
| *MRPL52* | ENST00000355151.5 | Down | 0.64 | chr14 | 23299452 | 23304158 | cds, utr3 | 6.31E-05 |
| *TATDN2* | ENST00000448281.2 | Down | 0.64 | chr3 | 10302021 | 10302351 | cds | 4.37E-03 |
| *ZSWIM8* | ENST00000605216.1 | Down | 0.64 | chr10 | 75552410 | 75553355 | cds | 3.16E-03 |
| *COQ10B* | ENST00000263960.2 | Down | 0.64 | chr2 | 198339136 | 198339555 | utr3 | 7.59E-03 |
| *PDCL* | ENST00000394285.3 | Down | 0.65 | chr9 | 125582822 | 125585435 | utr3, cds | 1.82E-02 |
| *PDGFB* | ENST00000381551.4 | Down | 0.65 | chr22 | 39620939 | 39621147 | utr3 | 1.23E-03 |
| *CLPX* | ENST00000300107.3 | Down | 0.65 | chr15 | 65441573 | 65442829 | utr3 | 2.00E-02 |
| *RSU1* | ENST00000602389.1 | Down | 0.65 | chr10 | 16632618 | 16633215 | utr3 | 1.17E-02 |
| *ALKBH4* | ENST00000292566.3 | Down | 0.65 | chr7 | 102097188 | 102097930 | utr3, cds | 7.08E-03 |
| *ZSWIM8* | ENST00000604524.1 | Down | 0.65 | chr10 | 75552403 | 75553348 | cds | 4.17E-03 |
| *CPSF3L* | ENST00000419704.1 | Down | 0.65 | chr1 | 1250817 | 1259973 | cds | 1.23E-03 |
| *TLK1* | ENST00000434911.2 | Down | 0.65 | chr2 | 171913031 | 171923453 | utr5, cds | 1.17E-04 |
| *EML3* | ENST00000531557.1 | Down | 0.66 | chr11 | 62375225 | 62376059 | cds | 4.07E-02 |
| *LIPT1* | ENST00000393471.2 | Down | 0.66 | chr2 | 99778951 | 99779611 | utr3, cds | 2.45E-02 |
| *ZSWIM8* | ENST00000398706.2 | Down | 0.66 | chr10 | 75552415 | 75553361 | cds | 4.57E-03 |
| *ZFYVE27* | ENST00000359980.3 | Down | 0.66 | chr10 | 99519303 | 99519603 | utr3 | 6.31E-03 |
| *RSU1* | ENST00000345264.5 | Down | 0.66 | chr10 | 16632609 | 16633209 | utr3 | 1.45E-02 |
| *ITSN1* | ENST00000381283.3 | Down | 0.66 | chr21 | 35144380 | 35144586 | cds | 5.50E-03 |
| *EML3* | ENST00000278845.4 | Down | 0.66 | chr11 | 62375224 | 62376058 | cds | 4.37E-02 |
| *DENND1A* | ENST00000542603.1 | Down | 0.66 | chr9 | 126165749 | 126213028 | cds | 1.62E-02 |
| *RSU1* | ENST00000377921.3 | Down | 0.66 | chr10 | 16632609 | 16633208 | utr3 | 1.51E-02 |
| *BMP6* | ENST00000283147.6 | Down | 0.66 | chr6 | 7880578 | 7880968 | utr3 | 6.03E-04 |
| *GMPR2* | ENST00000559943.1 | Down | 0.66 | chr14 | 24706508 | 24707972 | utr3, cds | 1.51E-03 |
| *ZFYVE27* | ENST00000423811.1 | Down | 0.66 | chr10 | 99519306 | 99519606 | utr3 | 7.41E-03 |
| *TMED1* | ENST00000214869.2 | Down | 0.66 | chr19 | 10943381 | 10943501 | utr3 | 2.45E-02 |
| *TCOF1* | ENST00000451292.1 | Down | 0.67 | chr5 | 149754566 | 149755357 | cds | 5.37E-03 |
| *SHC1* | ENST00000368450.1 | Down | 0.67 | chr1 | 154942563 | 154946792 | cds, utr5 | 2.24E-03 |
| *ZFYVE27* | ENST00000370610.3 | Down | 0.67 | chr10 | 99519293 | 99519592 | utr3 | 9.33E-03 |
| *ZFYVE27* | ENST00000356257.4 | Down | 0.67 | chr10 | 99519293 | 99519592 | utr3 | 9.33E-03 |
| *SHC1* | ENST00000448116.2 | Down | 0.67 | chr1 | 154942538 | 154942747 | cds | 8.91E-04 |
| *MRPL52* | ENST00000556840.1 | Down | 0.67 | chr14 | 23299448 | 23303556 | cds, utr3, utr5 | 6.03E-04 |
