# Supplementary material for: Integrated analysis of the transcriptome-wide m6A methylome in preeclampsia and healthy control placentas
Source: PeerJ. 2020 Sep 15;8:e9880. doi: 10.7717/peerj.9880 (PMC7500358; doi:10.7717/peerj.9880)
Supplement: Supplemental Information 4 [file peerj-08-9880-s004.docx]

**Table S4. The distribution of transcripts with a significant change in both m6A level and expression in preeclampsia.**

| **Gene Name** | **Log2 FC**  **(gene expression)** | **P-value of**  **gene expression** | **Log2 FC**  **(m6A methylation)** | **P-value of**  **m6A methylation** | **Quadrant** |
| --- | --- | --- | --- | --- | --- |
| *ANKRD49* | -1.51 | 4.63E-02 | 0.78 | 3.16E-02 | Hyper-down |
| *C5orf42* | -0.94 | 1.19E-02 | 0.60 | 1.18E-01 | Hyper-down |
| *CBFA2T2* | -0.70 | 1.37E-01 | 1.35 | 9.12E-02 | Hyper-down |
| *CFLAR* | -0.90 | 2.57E-01 | 1.19 | 2.24E-01 | Hyper-down |
| *COPRS* | -0.81 | 3.76E-01 | 0.59 | 3.80E-02 | Hyper-down |
| *HRH1* | -1.04 | 5.72E-02 | 1.42 | 5.75E-02 | Hyper-down |
| *HS3ST6* | -0.70 | 8.67E-03 | 1.22 | 1.51E-02 | Hyper-down |
| *JMJD1C* | -0.68 | 6.01E-01 | 0.77 | 1.00E-02 | Hyper-down |
| *MIOS* | -1.11 | 6.71E-02 | 0.71 | 5.62E-02 | Hyper-down |
| *NEK1* | -0.93 | 1.71E-02 | 0.90 | 1.20E-02 | Hyper-down |
| *PLEC* | -0.89 | 3.55E-01 | 1.74 | 1.67E-01 | Hyper-down |
| *PRKG1* | -0.59 | 1.28E-01 | 1.39 | 1.23E-01 | Hyper-down |
| *REPIN1* | -0.89 | 1.06E-01 | 0.82 | 2.00E-04 | Hyper-down |
| *REPIN1* | -0.66 | 1.44E-01 | 0.83 | 1.82E-04 | Hyper-down |
| *RRM2* | -0.72 | 1.83E-01 | 2.32 | 1.85E-01 | Hyper-down |
| *SH3YL1* | -0.67 | 2.84E-01 | 1.69 | 1.76E-01 | Hyper-down |
| *SLIT2* | -0.90 | 3.04E-01 | 0.77 | 2.04E-02 | Hyper-down |
| *SMG1* | -0.68 | 3.73E-01 | 0.84 | 2.87E-01 | Hyper-down |
| *TDRD3* | -0.59 | 3.45E-01 | 0.64 | 1.29E-01 | Hyper-down |
| *TSPAN14* | -0.77 | 2.01E-01 | 3.49 | 6.76E-02 | Hyper-down |
| *UBE4A* | -1.28 | 5.78E-02 | 0.67 | 3.63E-02 | Hyper-down |
| *ZFX* | -1.54 | 9.55E-02 | 0.92 | 2.63E-03 | Hyper-down |
| *ZNF146* | -1.94 | 1.07E-01 | 0.59 | 5.62E-03 | Hyper-down |
| *ZNF605* | -0.97 | 1.28E-01 | 0.64 | 2.19E-02 | Hyper-down |
| *ZNF845* | -0.76 | 1.23E-01 | 1.38 | 1.17E-01 | Hyper-down |
| *LDLR* | 0.91 | 1.61E-01 | 1.23 | 2.48E-01 | Hyper-up |
| *APC* | 0.92 | 1.22E-01 | 0.66 | 1.28E-01 | Hyper-up |
| *SBNO1* | 0.64 | 2.27E-01 | 0.69 | 1.83E-01 | Hyper-up |
| *PLEKHA6* | 0.78 | 5.97E-01 | 0.90 | 1.51E-03 | Hyper-up |
| *TNFRSF10B* | 0.97 | 6.84E-02 | 1.17 | 1.17E-03 | Hyper-up |
| *MBNL1* | 0.79 | 1.53E-01 | 1.26 | 3.24E-01 | Hyper-up |
| *CLSPN* | 0.66 | 7.92E-02 | 2.46 | 1.15E-01 | Hyper-up |
| *POLE* | 0.68 | 3.00E-01 | 0.74 | 5.01E-02 | Hyper-up |
| *PLA2G16* | 0.86 | 8.97E-02 | 0.87 | 2.95E-02 | Hyper-up |
| *PRX* | 0.59 | 2.36E-01 | 1.14 | 3.24E-02 | Hyper-up |
| *FAM174B* | 0.68 | 2.72E-02 | 1.02 | 6.03E-02 | Hyper-up |
| *MKLN1* | 1.11 | 8.47E-02 | 1.07 | 5.50E-03 | Hyper-up |
| *PLEC* | 1.09 | 4.58E-01 | 1.84 | 1.48E-01 | Hyper-up |
| *PLEC* | 1.05 | 6.05E-02 | 1.76 | 1.64E-01 | Hyper-up |
| *ELK4* | 0.78 | 2.63E-01 | 0.64 | 1.32E-01 | Hyper-up |
| *TCF7L2* | 0.87 | 1.50E-01 | 0.74 | 3.16E-02 | Hyper-up |
| *ENDOG* | 0.65 | 6.42E-03 | 0.87 | 3.80E-03 | Hyper-up |
| *ZFX* | 1.22 | 2.21E-01 | 0.93 | 1.35E-03 | Hyper-up |
| *ARSK* | 0.77 | 1.22E-01 | 1.74 | 3.31E-02 | Hyper-up |
| *GALNT3* | 0.78 | 3.41E-02 | 2.81 | 2.37E-01 | Hyper-up |
| *JMJD1C* | 0.59 | 5.82E-01 | 0.73 | 8.51E-03 | Hyper-up |
| *DST* | 0.86 | 1.53E-01 | 0.81 | 3.72E-02 | Hyper-up |
| *MEMO1* | 0.97 | 2.20E-01 | 0.73 | 1.53E-01 | Hyper-up |
| *ARAP1* | 0.77 | 1.52E-01 | 0.71 | 1.86E-02 | Hyper-up |
| *UBE4A* | 2.49 | 2.52E-02 | 0.67 | 3.80E-02 | Hyper-up |
| *LRCH3* | 0.61 | 2.73E-01 | 0.85 | 2.88E-01 | Hyper-up |
| *PAQR8* | 1.05 | 6.91E-02 | 1.80 | 3.55E-02 | Hyper-up |
| *AP1M1* | 0.60 | 5.72E-01 | 0.77 | 1.32E-02 | Hyper-up |
| *ZNF146* | 1.64 | 1.20E-01 | 0.59 | 5.37E-03 | Hyper-up |
| *MSH6* | 0.90 | 4.08E-01 | 0.65 | 1.38E-04 | Hyper-up |
| *COBL* | -0.88 | 5.06E-02 | -0.71 | 1.86E-02 | Hypo-down |
| *TTBK2* | -0.65 | 6.32E-02 | -0.60 | 1.02E-01 | Hypo-down |
| *CFLAR* | -0.90 | 2.57E-01 | -0.90 | 1.17E-03 | Hypo-down |
| *C11orf54* | -0.95 | 1.28E-01 | -0.70 | 4.90E-02 | Hypo-down |
| *YME1L1* | -1.17 | 4.78E-02 | -0.78 | 8.91E-07 | Hypo-down |
| *ZFX* | -1.54 | 9.55E-02 | -0.66 | 9.55E-02 | Hypo-down |
| *TRAF3IP1* | -0.67 | 2.26E-02 | -1.45 | 2.02E-01 | Hypo-down |
| *UBFD1* | -0.74 | 4.24E-01 | -0.80 | 1.58E-03 | Hypo-down |
| *PLEC* | -0.89 | 3.55E-01 | -2.58 | 2.42E-01 | Hypo-down |
| *SMAD9* | -0.98 | 8.16E-02 | -1.53 | 5.62E-02 | Hypo-down |
| *MOGS* | -0.99 | 3.67E-01 | -0.67 | 2.69E-03 | Hypo-down |
| *KLHL8* | -0.60 | 5.06E-01 | -0.96 | 8.91E-02 | Hypo-down |
| *TLK1* | -1.17 | 1.01E-01 | -0.61 | 1.17E-04 | Hypo-down |
| *ENTPD1* | -0.72 | 2.48E-01 | -1.44 | 9.55E-02 | Hypo-down |
| *GMPPB* | -0.80 | 1.45E-01 | -1.04 | 1.86E-01 | Hypo-down |
| *UBFD1* | -0.71 | 3.56E-01 | -0.69 | 6.76E-03 | Hypo-down |
| *CDK19* | 0.68 | 3.28E-02 | -0.70 | 9.12E-02 | Hypo-up |
| *CILP* | 0.87 | 2.61E-01 | -2.51 | 7.76E-02 | Hypo-up |
| *CNEP1R1* | 0.76 | 4.22E-01 | -0.83 | 3.63E-02 | Hypo-up |
| *COL18A1* | 1.02 | 1.31E-01 | -1.92 | 1.24E-01 | Hypo-up |
| *CUL9* | 0.76 | 2.84E-02 | -0.66 | 6.61E-02 | Hypo-up |
| *DSTYK* | 0.59 | 2.09E-01 | -0.97 | 1.76E-01 | Hypo-up |
| *ELK4* | 0.78 | 2.63E-01 | -1.08 | 5.62E-02 | Hypo-up |
| *ENTPD1* | 1.18 | 4.58E-01 | -1.74 | 1.00E-01 | Hypo-up |
| *IGSF10* | 0.67 | 3.05E-01 | -2.14 | 1.64E-01 | Hypo-up |
| *INTS11* | 0.87 | 1.71E-01 | -0.62 | 1.23E-03 | Hypo-up |
| *LIMS1* | 0.85 | 1.92E-01 | -0.76 | 3.47E-03 | Hypo-up |
| *LRCH3* | 0.61 | 2.73E-01 | -1.85 | 4.57E-02 | Hypo-up |
| *NPFFR2* | 1.34 | 1.78E-01 | -2.00 | 2.14E-01 | Hypo-up |
| *PLAC8* | 1.82 | 2.03E-02 | -2.26 | 9.55E-02 | Hypo-up |
| *PLEC* | 1.09 | 4.58E-01 | -2.72 | 1.53E-01 | Hypo-up |
| *PLEC* | 1.05 | 6.05E-02 | -2.91 | 1.37E-01 | Hypo-up |
| *PSD4* | 1.05 | 2.86E-02 | -1.19 | 7.94E-02 | Hypo-up |
| *REV3L* | 1.28 | 1.37E-01 | -0.89 | 8.71E-02 | Hypo-up |
| *RGPD1* | 0.66 | 1.81E-01 | -1.46 | 3.16E-01 | Hypo-up |
| *RPL7* | 1.13 | 5.04E-02 | -1.06 | 3.37E-01 | Hypo-up |
| *RRP1B* | 0.62 | 7.46E-02 | -0.83 | 6.61E-02 | Hypo-up |
| *RSU1* | 1.00 | 4.45E-02 | -0.60 | 1.51E-02 | Hypo-up |
| *SMAD9* | 0.68 | 2.09E-01 | -1.53 | 5.62E-02 | Hypo-up |
| *SMG1* | 0.77 | 1.84E-01 | -0.67 | 2.32E-01 | Hypo-up |
| *SNRNP25* | 0.82 | 4.16E-02 | -0.88 | 5.25E-02 | Hypo-up |
| *SPATS2* | 0.75 | 1.89E-01 | -2.46 | 3.30E-01 | Hypo-up |
| *SSH1* | 0.83 | 1.68E-01 | -0.82 | 2.82E-02 | Hypo-up |
| *STAU1* | 1.41 | 8.57E-02 | -1.02 | 1.61E-01 | Hypo-up |
| *UGDH* | 1.96 | 1.60E-01 | -0.83 | 1.95E-02 | Hypo-up |
| *WDR44* | 1.09 | 9.81E-02 | -0.88 | 5.13E-02 | Hypo-up |
| *YME1L1* | 2.66 | 1.34E-02 | -0.76 | 9.77E-07 | Hypo-up |
| *ZFX* | 1.22 | 2.21E-01 | -0.62 | 1.09E-01 | Hypo-up |
| *ZNF511* | 0.63 | 6.19E-02 | -0.73 | 5.25E-02 | Hypo-up |
| *ZSWIM8* | 0.91 | 5.24E-02 | -0.62 | 4.17E-03 | Hypo-up |

FC: fold change; Hyper-down: hyper-methylated and down-regulated; Hyper-up: hyper-methylated and up-regulated; Hypo-down: Hypo-methylated and down-regulated; Hypo-up: hypo-methylated and up-regulated.
