# Supplementary figures and images for: Integrated analysis of the transcriptome-wide m6A methylome in preeclampsia and healthy control placentas
Source: PeerJ. 2020 Sep 15;8:e9880. doi: 10.7717/peerj.9880 (PMC7500358; doi:10.7717/peerj.9880)

Preeclampsia

Control

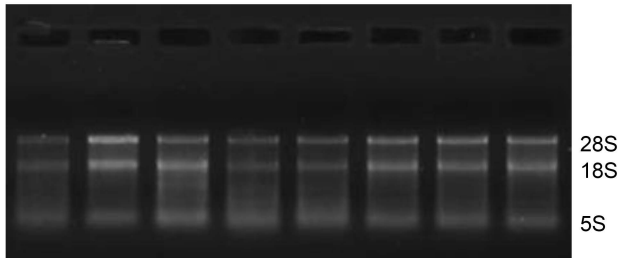

Supplement: Supplemental Information 5 [file peerj-08-9880-s005.pdf]

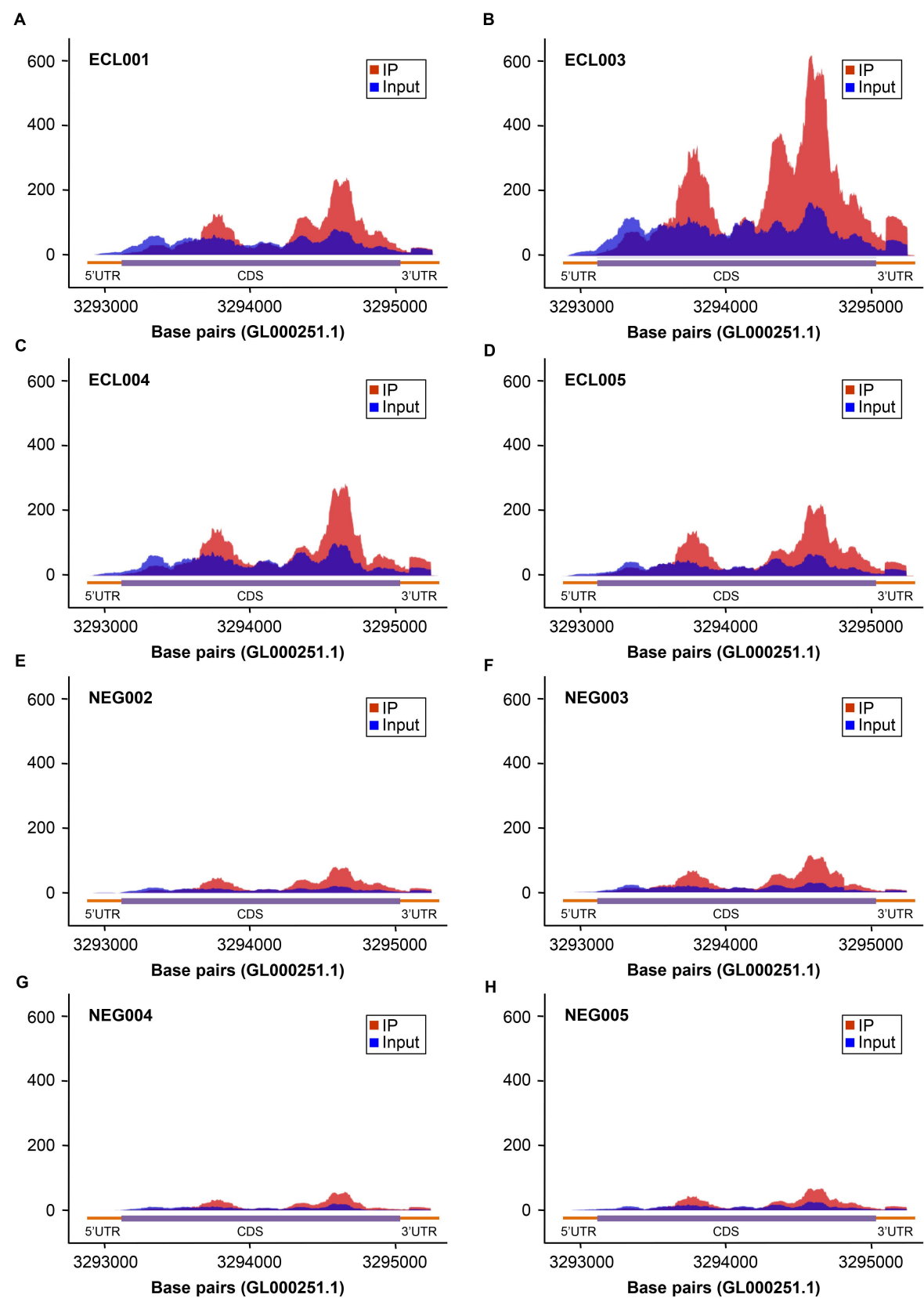

Supplement: Supplemental Information 6 — ECL001 (A), ECL003 (B), ECL004 (C), ECL005 (D). (G) Data visualisation analysis of HSPA1A mRNA m6A modifications in the control subjects: NEG002 (E), NEG003 (F), NEG004 (G), NEG005 (H). [file peerj-08-9880-s006.pdf]

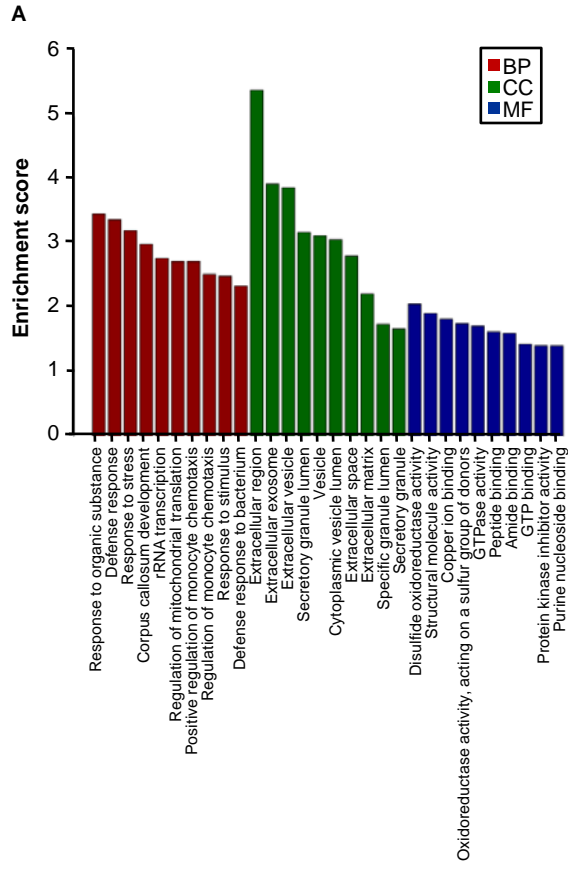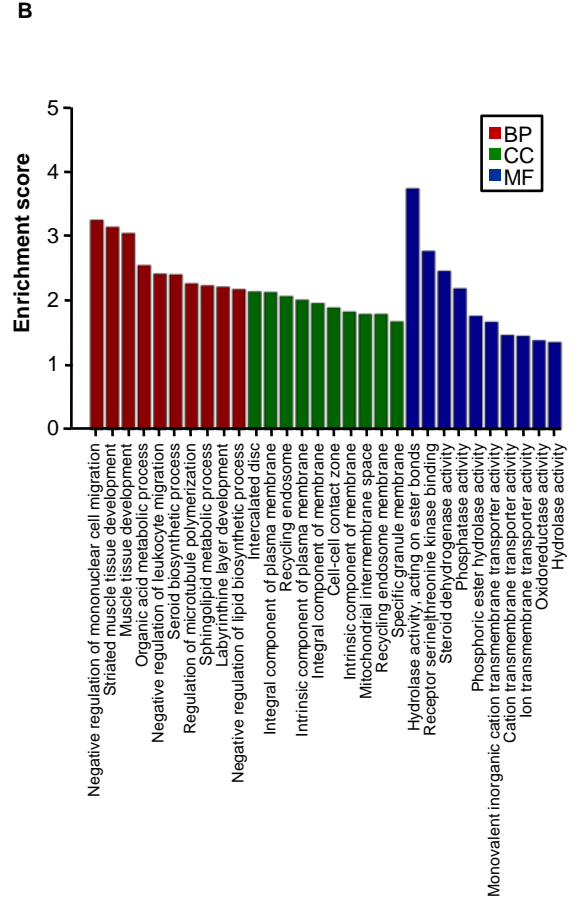

**C**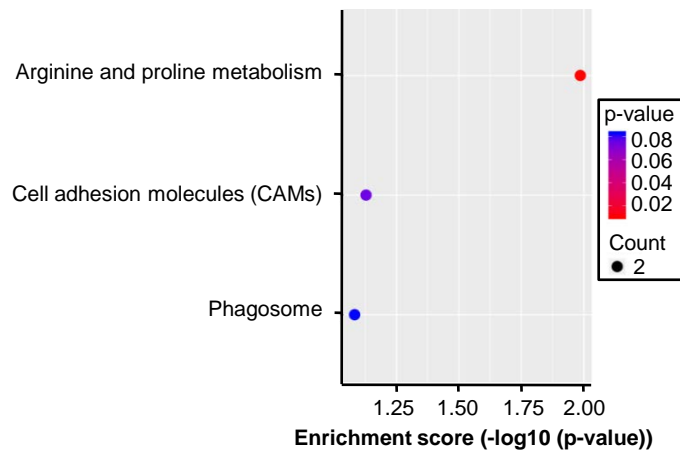**D**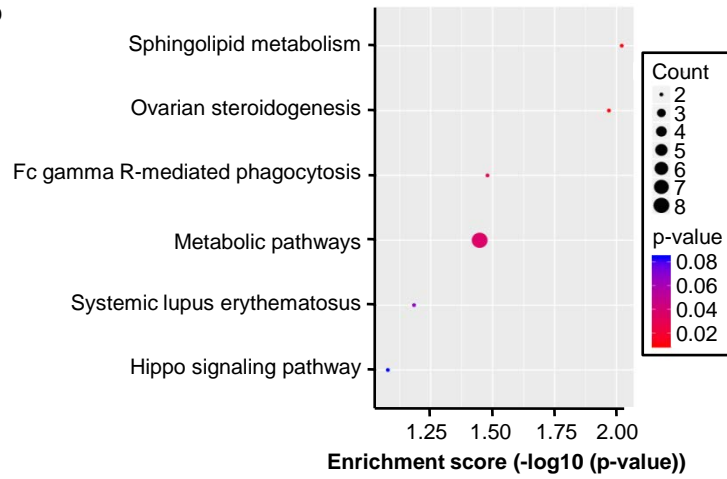

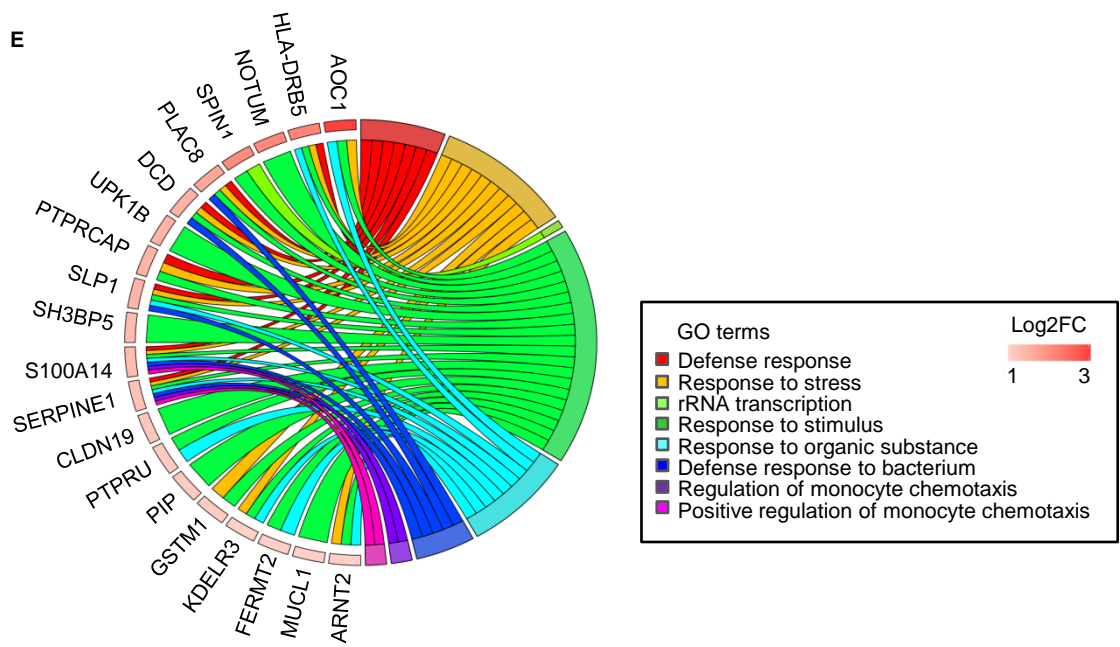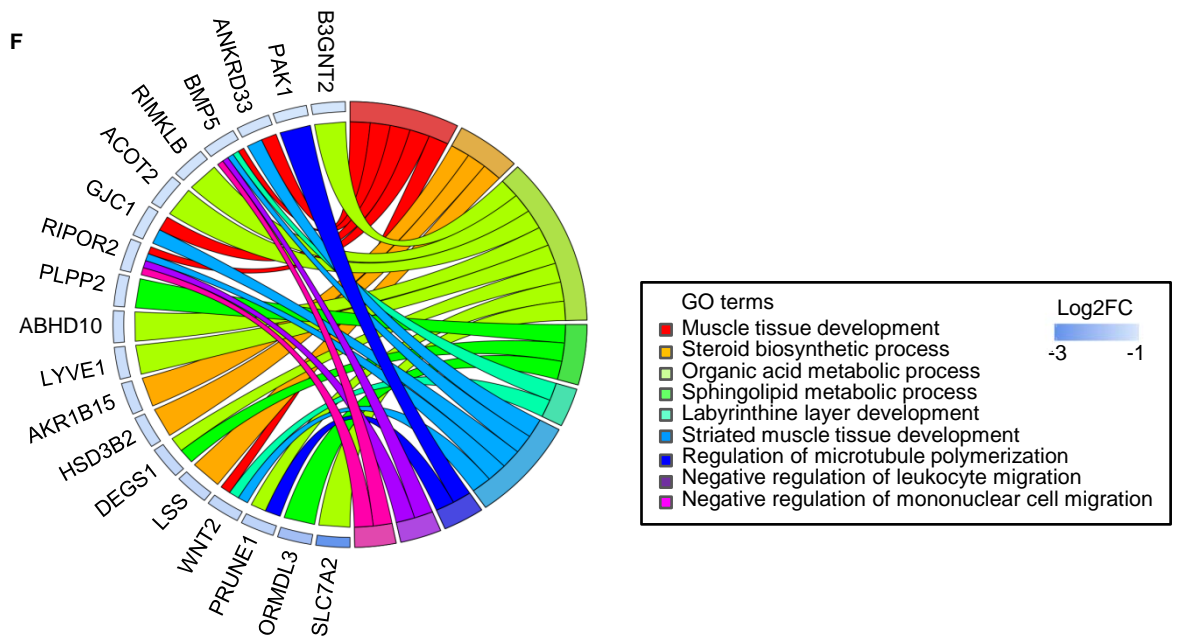

Supplement: Supplemental Information 7 — (A) Major enriched and significant GO-assessed up-regulated mRNAs. (B) Major enriched and significant GO-assessed down-regulated mRNAs. (C) The top ten significantly enriched pathways for the up-regulated mRNAs. (D) The top ten significantly enriched pathways for the down-regulated mRNAs. (E) The top 8 GO terms of biological process were significantly enriched for the 20 up-regulated genes in preeclampsia. (F) The top 9 GO terms of biological process were significantly enriched for the 19 down-regulated genes in preeclampsia. GO: gene ontology, KEGG: Kyoto encyclopedia of genes and genomes. [file peerj-08-9880-s007.pdf]

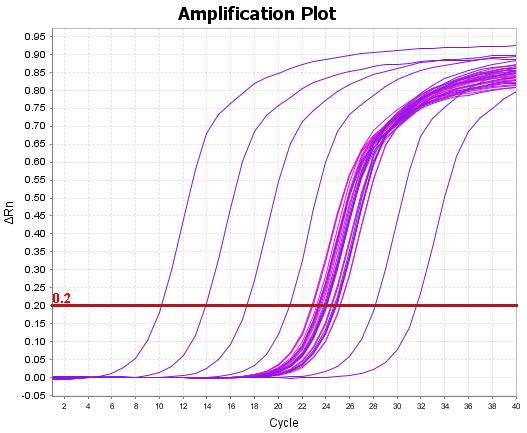

Supplement: Supplemental Information 8 [file peerj-08-9880-s008.zip › raw data/3.Amplification Plot-qRT-PCR/Amplification Plot ALKBH5.png]

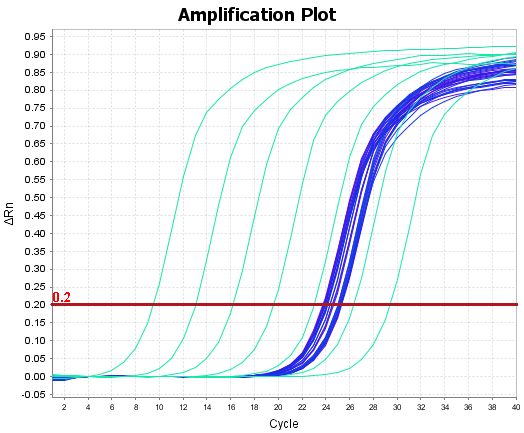

Supplement: Supplemental Information 8 [file peerj-08-9880-s008.zip › raw data/3.Amplification Plot-qRT-PCR/Amplification Plot FTO.png]

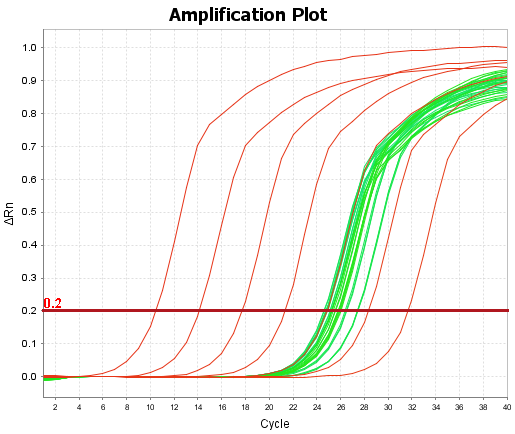

Supplement: Supplemental Information 8 [file peerj-08-9880-s008.zip › raw data/3.Amplification Plot-qRT-PCR/Amplification Plot Mettl14.png]

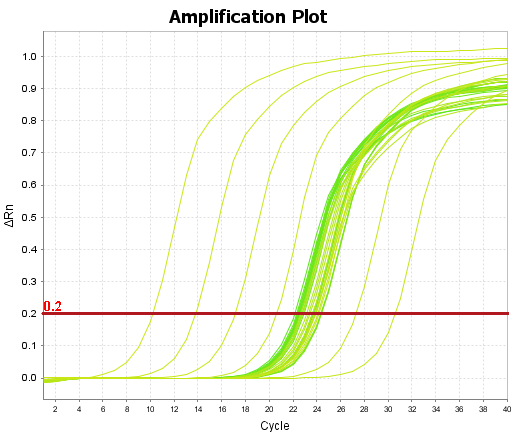

Supplement: Supplemental Information 8 [file peerj-08-9880-s008.zip › raw data/3.Amplification Plot-qRT-PCR/Amplification Plot Mettl3.png]

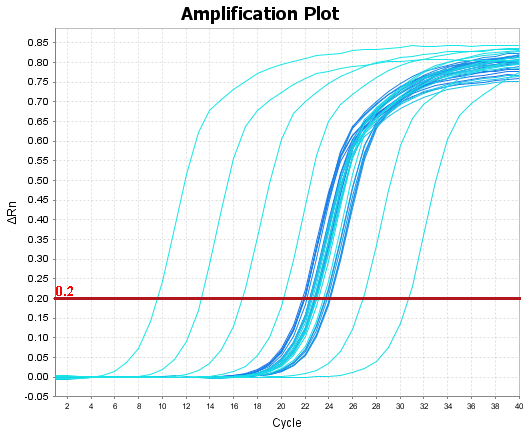

Supplement: Supplemental Information 8 [file peerj-08-9880-s008.zip › raw data/3.Amplification Plot-qRT-PCR/Amplification Plot WTAP.png]

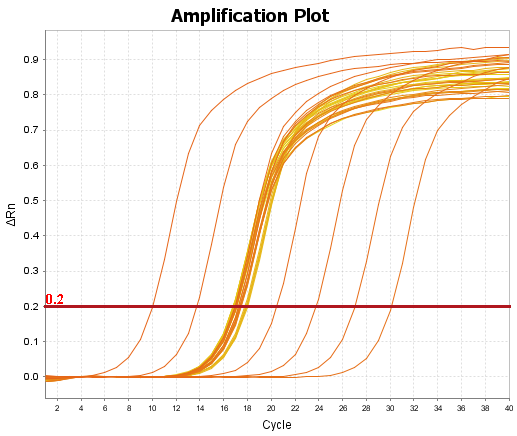

Supplement: Supplemental Information 8 [file peerj-08-9880-s008.zip › raw data/3.Amplification Plot-qRT-PCR/Amplification Plot a┬-actin.png]

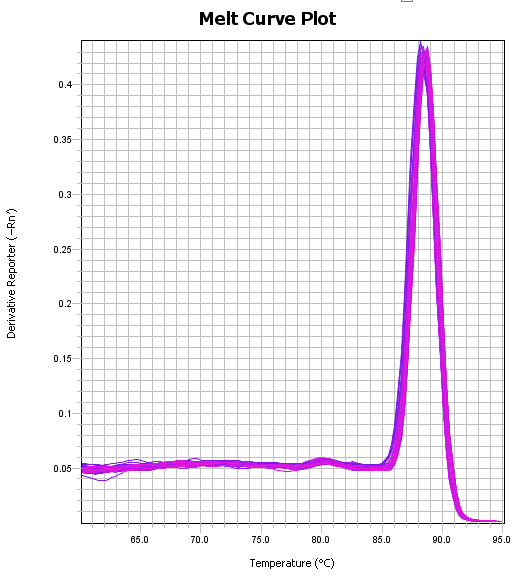

Supplement: Supplemental Information 8 [file peerj-08-9880-s008.zip › raw data/4.Melt Curve Plot-qRT-PCR/Melt Curve Plot ALKBH5.png]

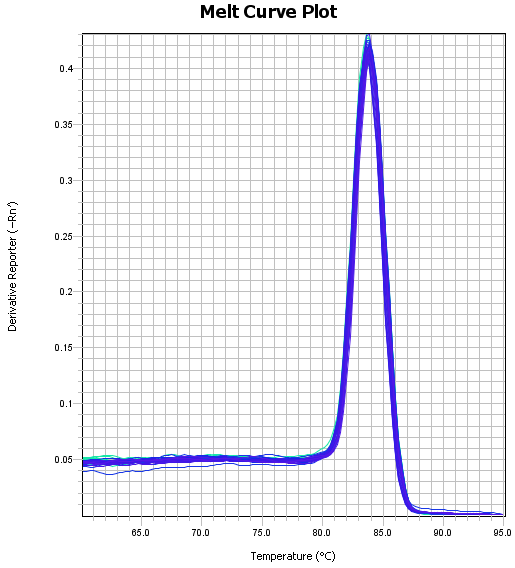

Supplement: Supplemental Information 8 [file peerj-08-9880-s008.zip › raw data/4.Melt Curve Plot-qRT-PCR/Melt Curve Plot FTO.png]

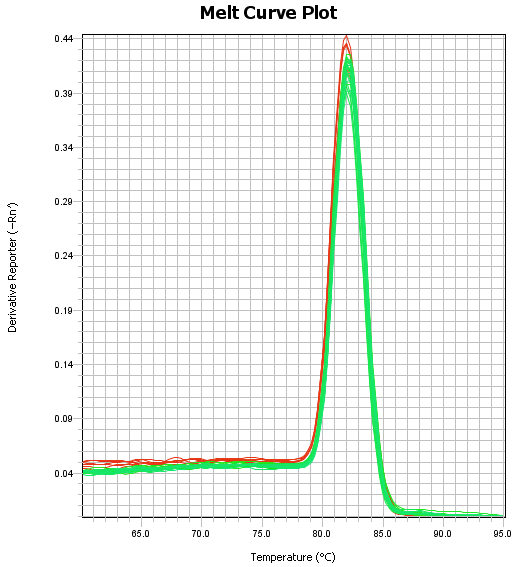

Supplement: Supplemental Information 8 [file peerj-08-9880-s008.zip › raw data/4.Melt Curve Plot-qRT-PCR/Melt Curve Plot Mettl14.png]

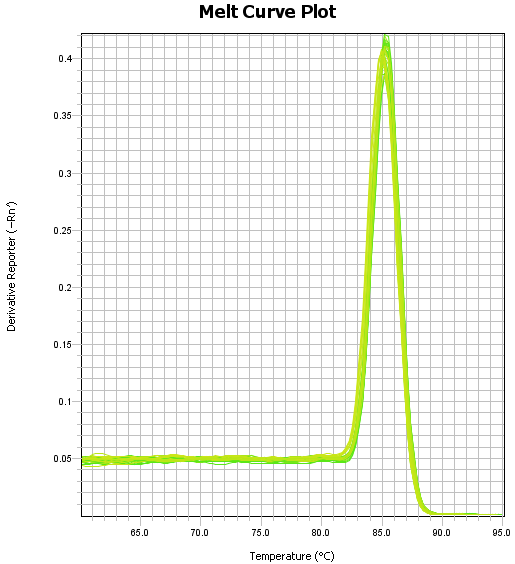

Supplement: Supplemental Information 8 [file peerj-08-9880-s008.zip › raw data/4.Melt Curve Plot-qRT-PCR/Melt Curve Plot Mettl3.png]

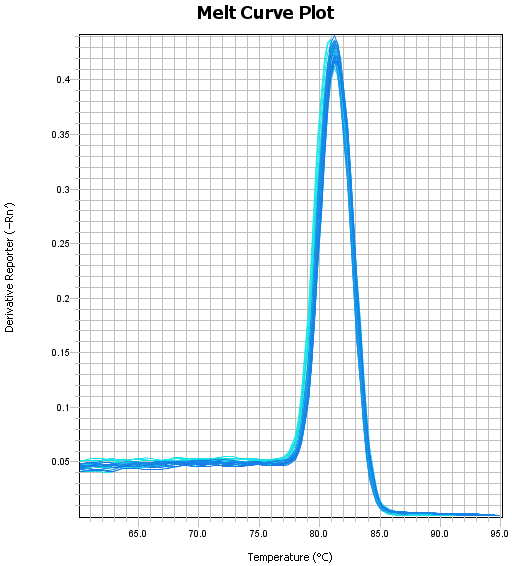

Supplement: Supplemental Information 8 [file peerj-08-9880-s008.zip › raw data/4.Melt Curve Plot-qRT-PCR/Melt Curve Plot WTAP.png]

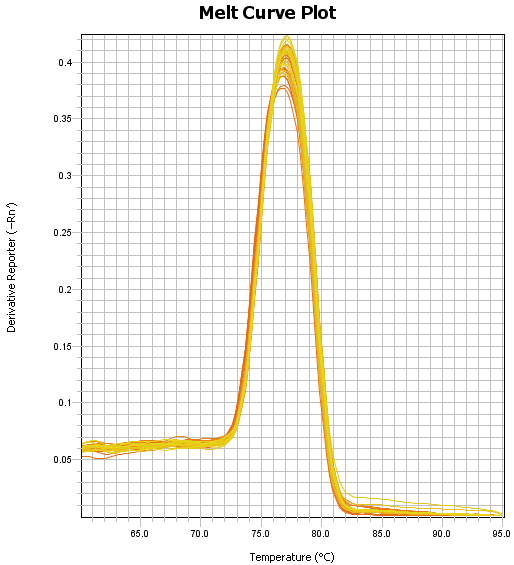

Supplement: Supplemental Information 8 [file peerj-08-9880-s008.zip › raw data/4.Melt Curve Plot-qRT-PCR/Melt Curve Plot a┬-actin.png]

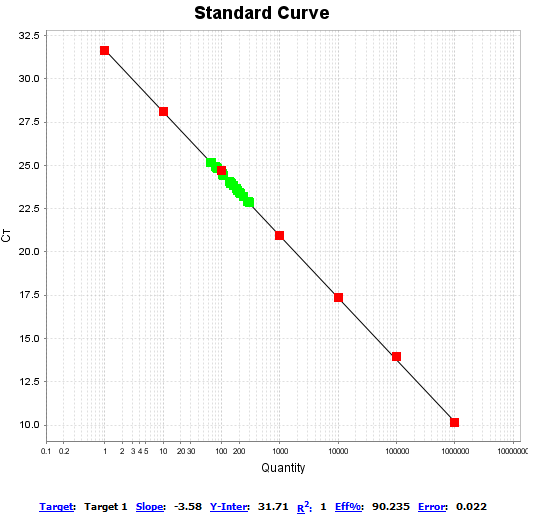

Supplement: Supplemental Information 8 [file peerj-08-9880-s008.zip › raw data/5.Standard Curve-qRT-PCR/Standard Curve ALKBH5.png]

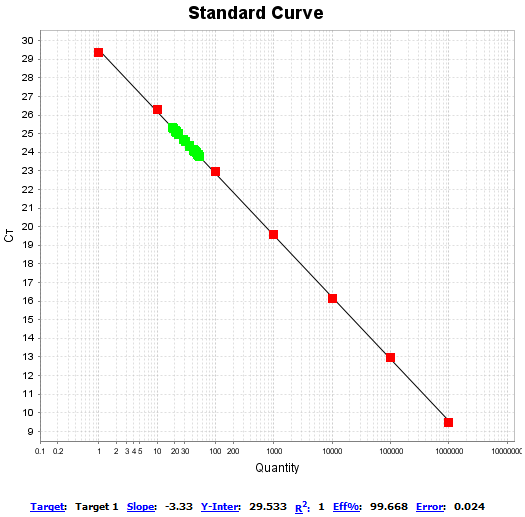

Supplement: Supplemental Information 8 [file peerj-08-9880-s008.zip › raw data/5.Standard Curve-qRT-PCR/Standard Curve FTO.png]

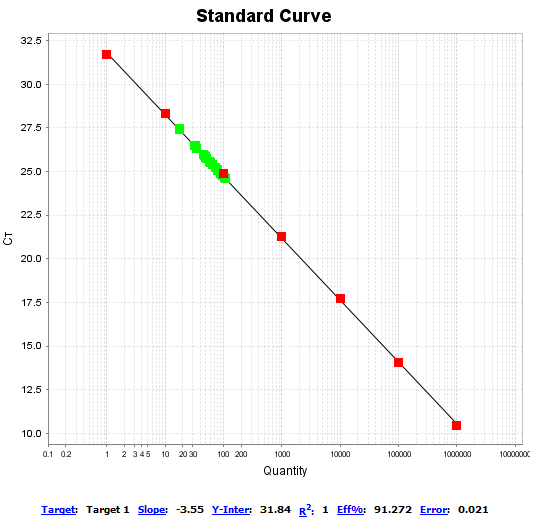

Supplement: Supplemental Information 8 [file peerj-08-9880-s008.zip › raw data/5.Standard Curve-qRT-PCR/Standard Curve Mettl14.png]

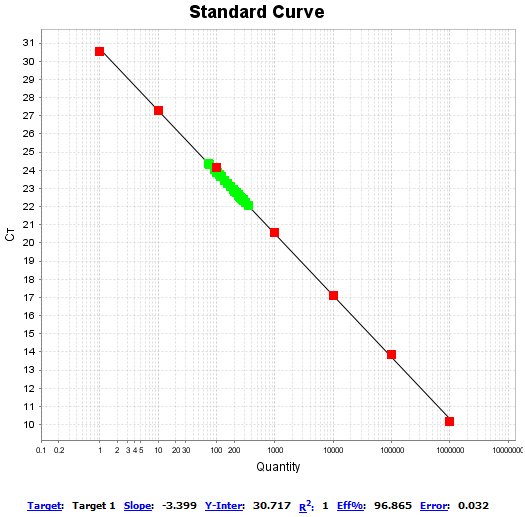

Supplement: Supplemental Information 8 [file peerj-08-9880-s008.zip › raw data/5.Standard Curve-qRT-PCR/Standard Curve Mettl3.png]

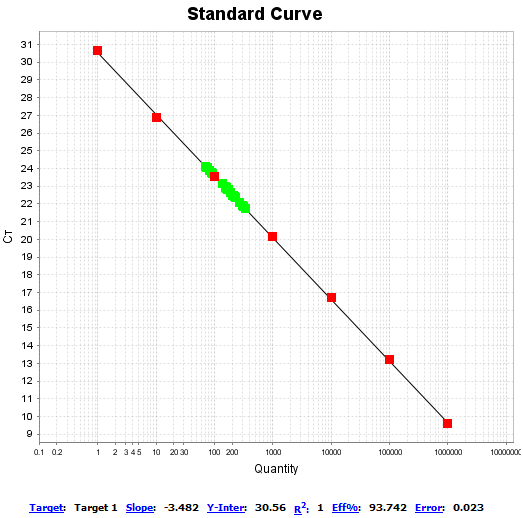

Supplement: Supplemental Information 8 [file peerj-08-9880-s008.zip › raw data/5.Standard Curve-qRT-PCR/Standard Curve WTAP.png]

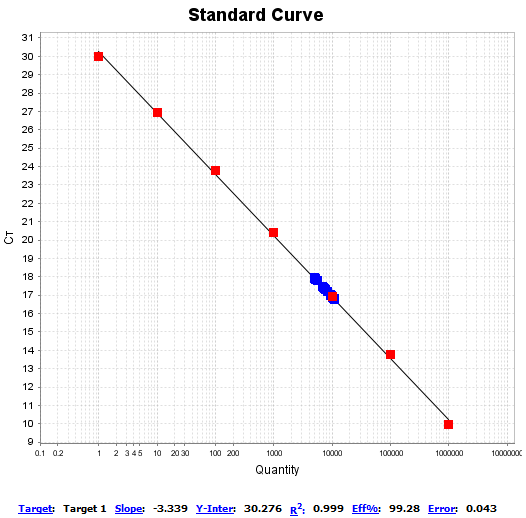

Supplement: Supplemental Information 8 [file peerj-08-9880-s008.zip › raw data/5.Standard Curve-qRT-PCR/Standard Curve a┬-actin.png]

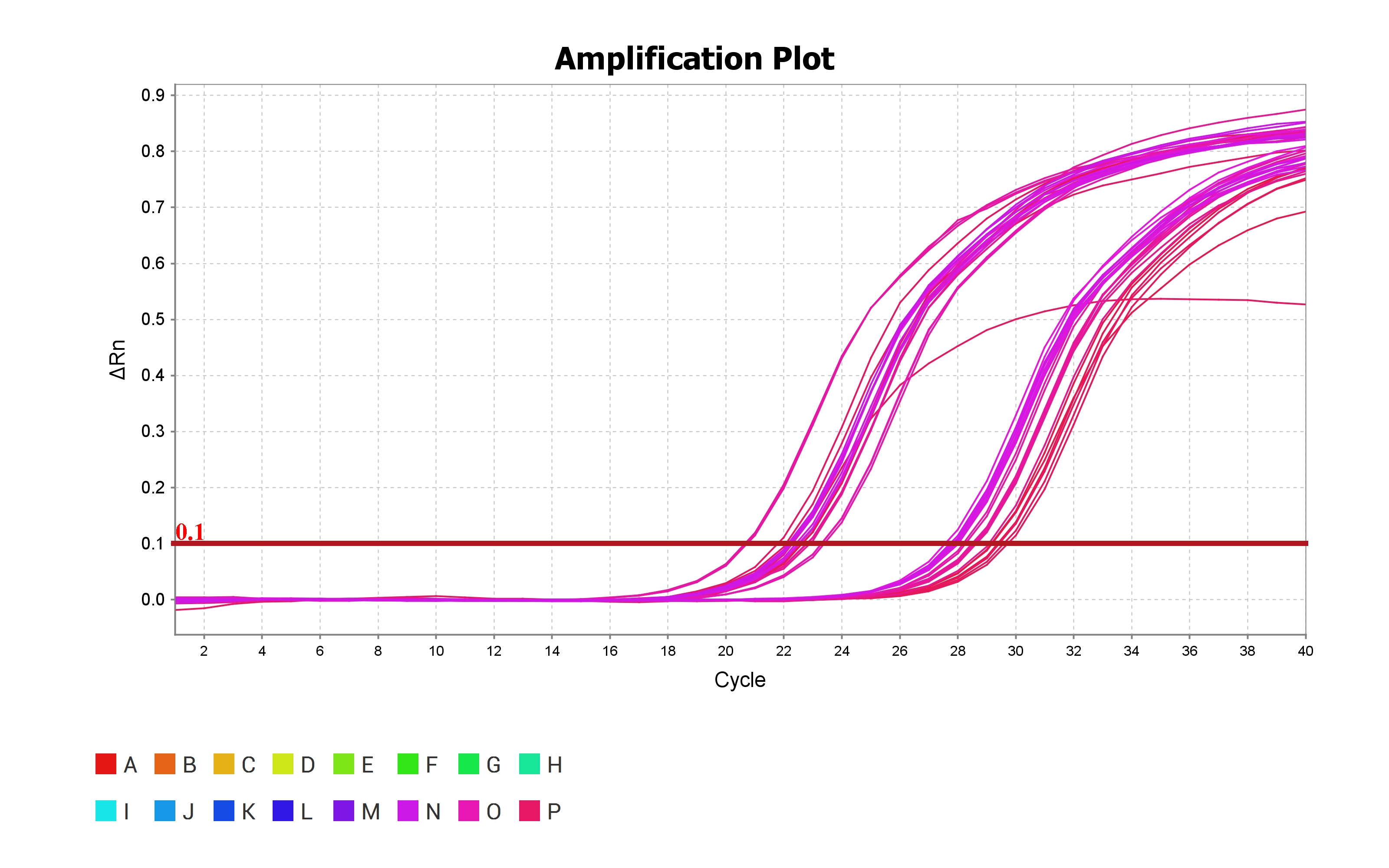

Supplement: Supplemental Information 9 [file peerj-08-9880-s009.zip › raw data-verify/1.RAW DATA-MazF-qPCR/5.Amplification Plot HSPA1A.jpg]

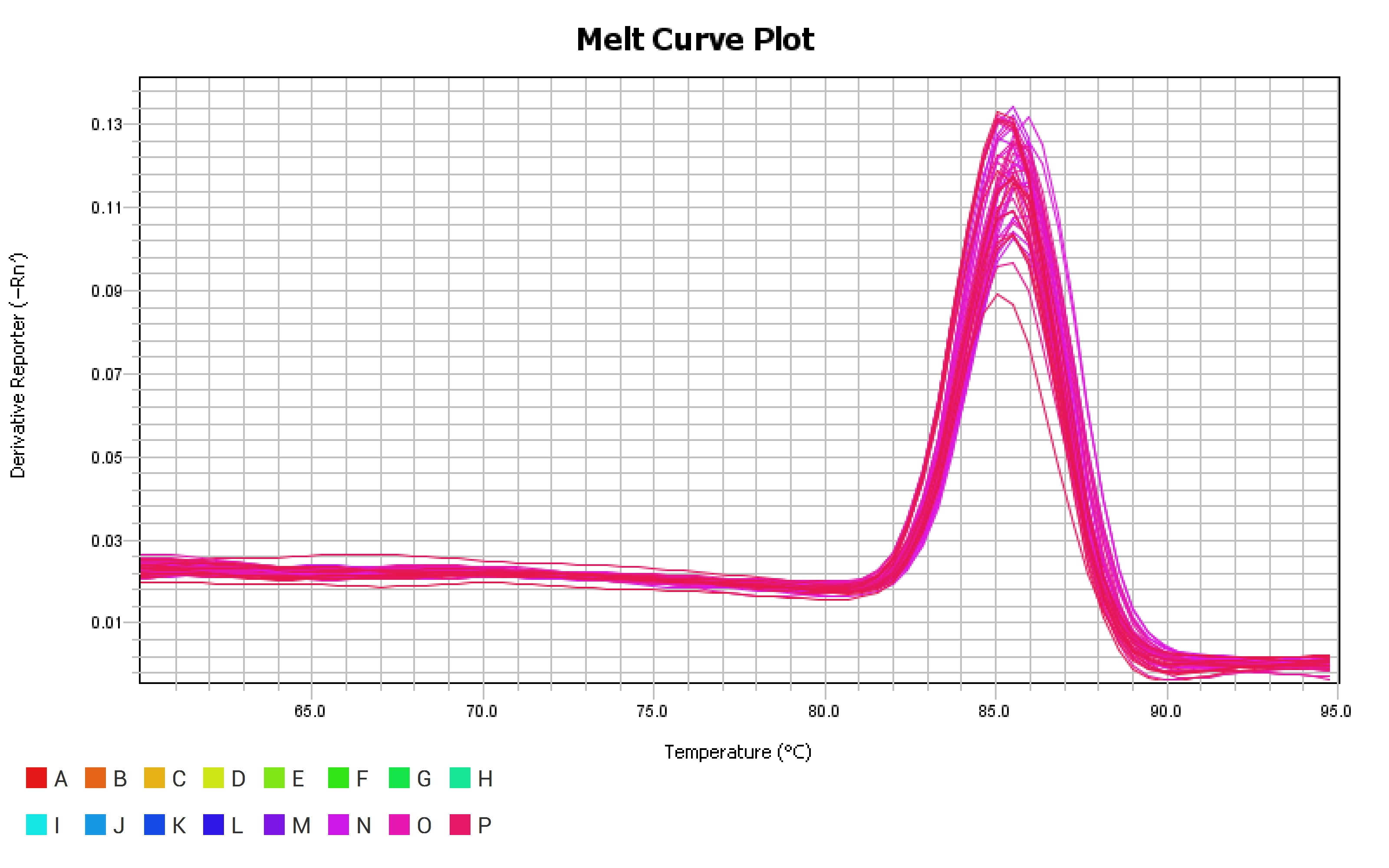

Supplement: Supplemental Information 9 [file peerj-08-9880-s009.zip › raw data-verify/1.RAW DATA-MazF-qPCR/6.Melt Curve Plot HSPA1A.jpg]

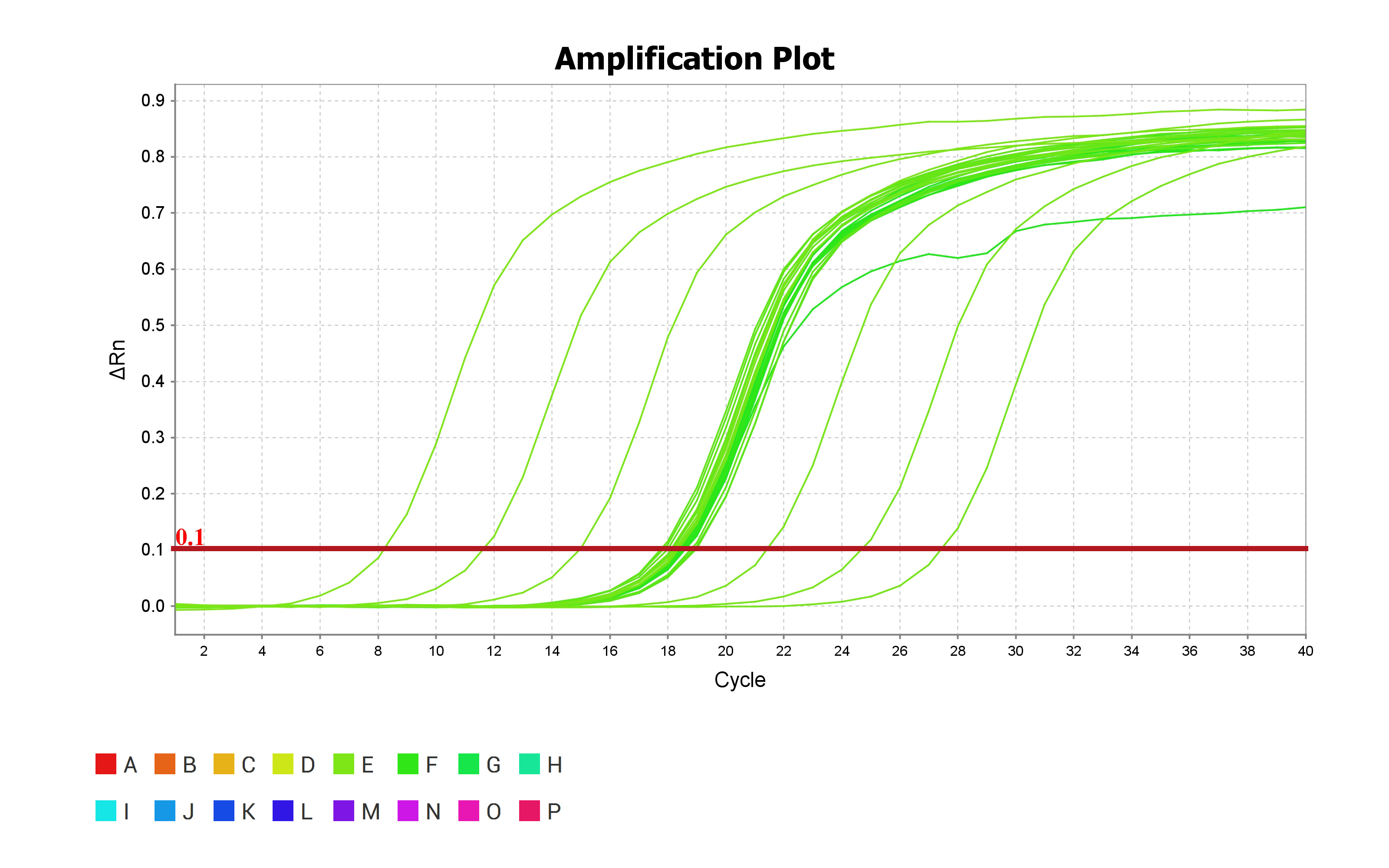

Supplement: Supplemental Information 9 [file peerj-08-9880-s009.zip › raw data-verify/2.RAW DATA-qRT-PCR(HSPA1Aú⌐/3.Amplification Plot-qRT-PCR/Amplification Plot ACTB.jpg]

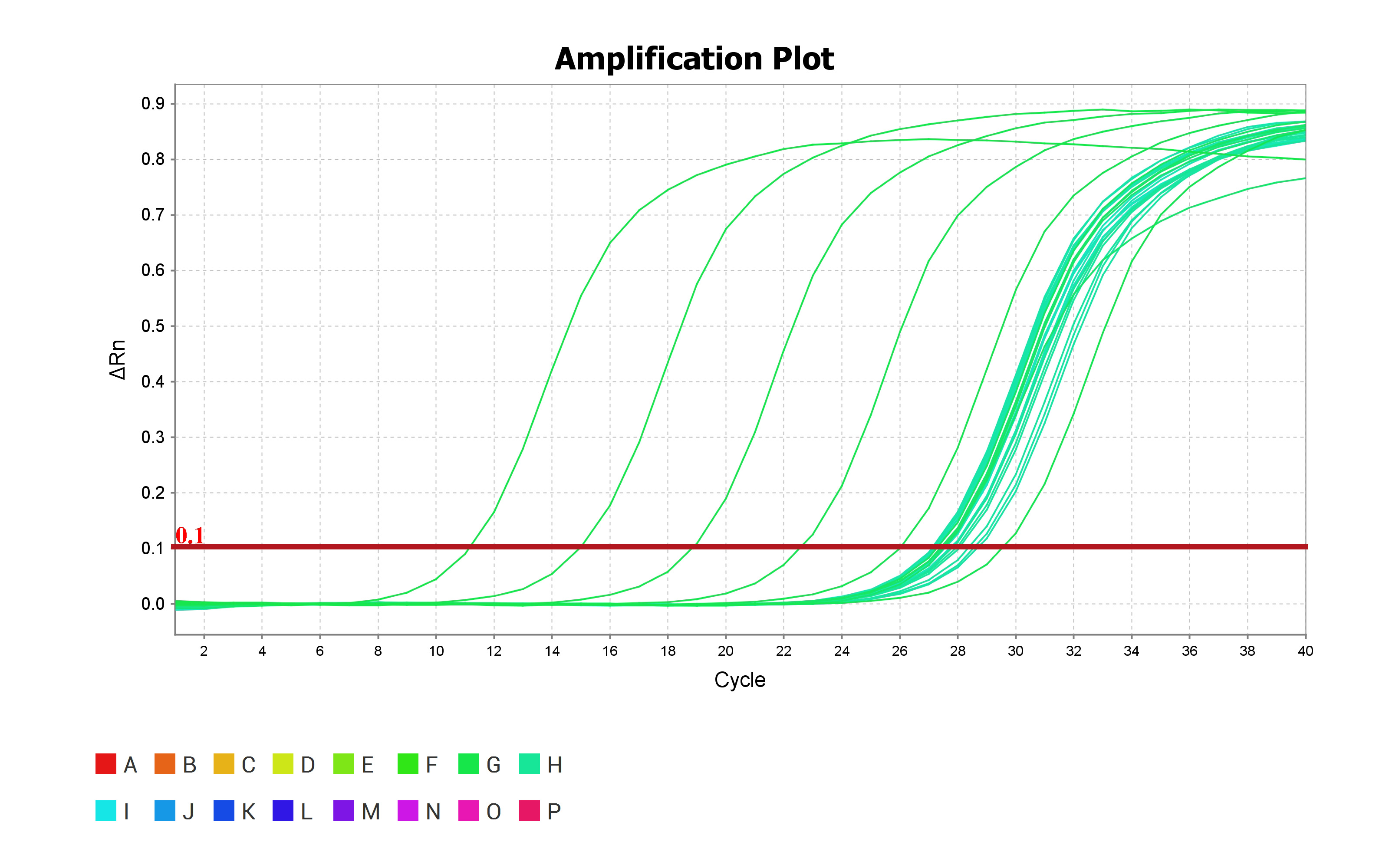

Supplement: Supplemental Information 9 [file peerj-08-9880-s009.zip › raw data-verify/2.RAW DATA-qRT-PCR(HSPA1Aú⌐/3.Amplification Plot-qRT-PCR/Amplification Plot HSPA1A.jpg]

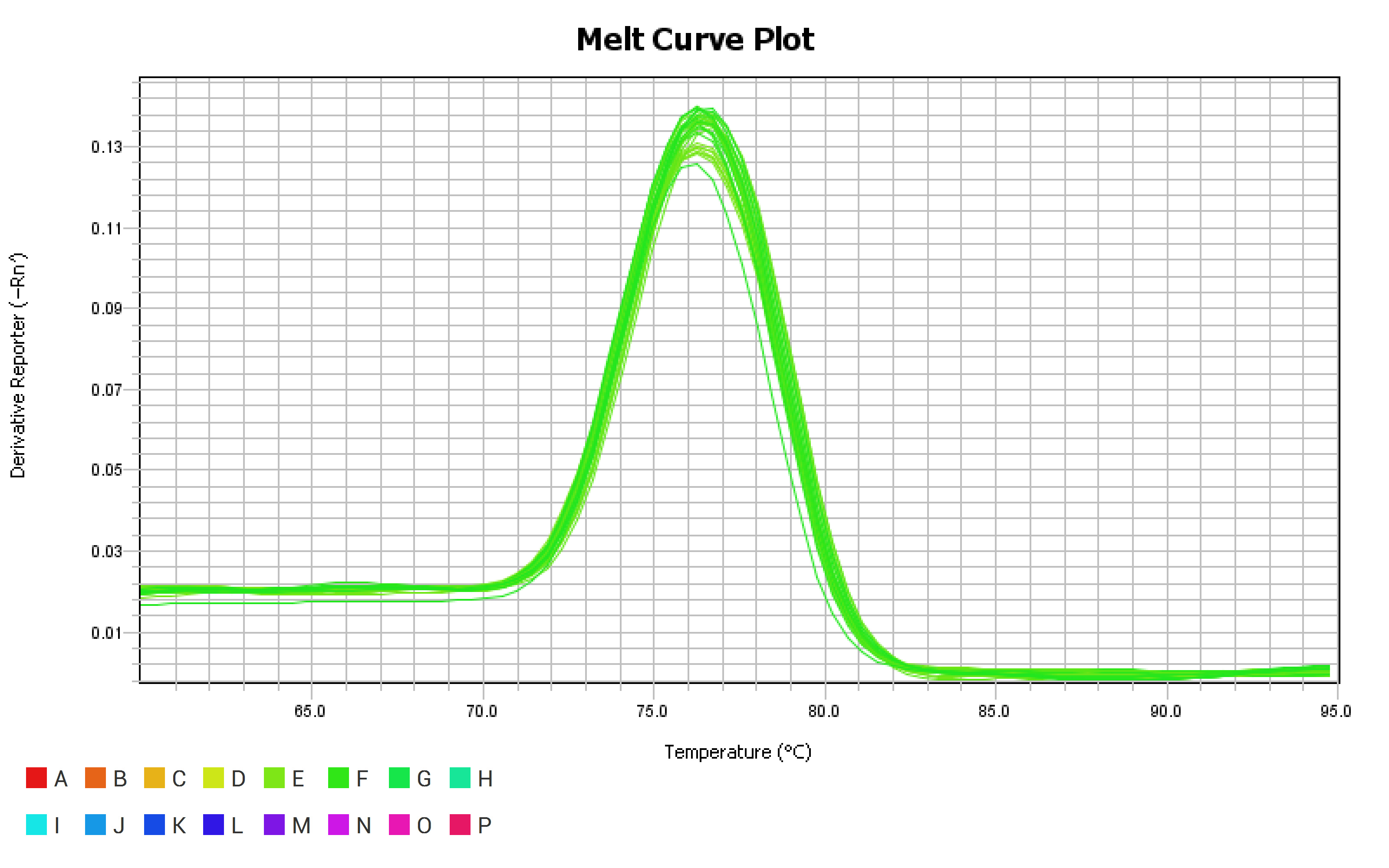

Supplement: Supplemental Information 9 [file peerj-08-9880-s009.zip › raw data-verify/2.RAW DATA-qRT-PCR(HSPA1Aú⌐/4.Melt Curve Plot-qRT-PCR/Melt Curve Plot ACTB.jpg]

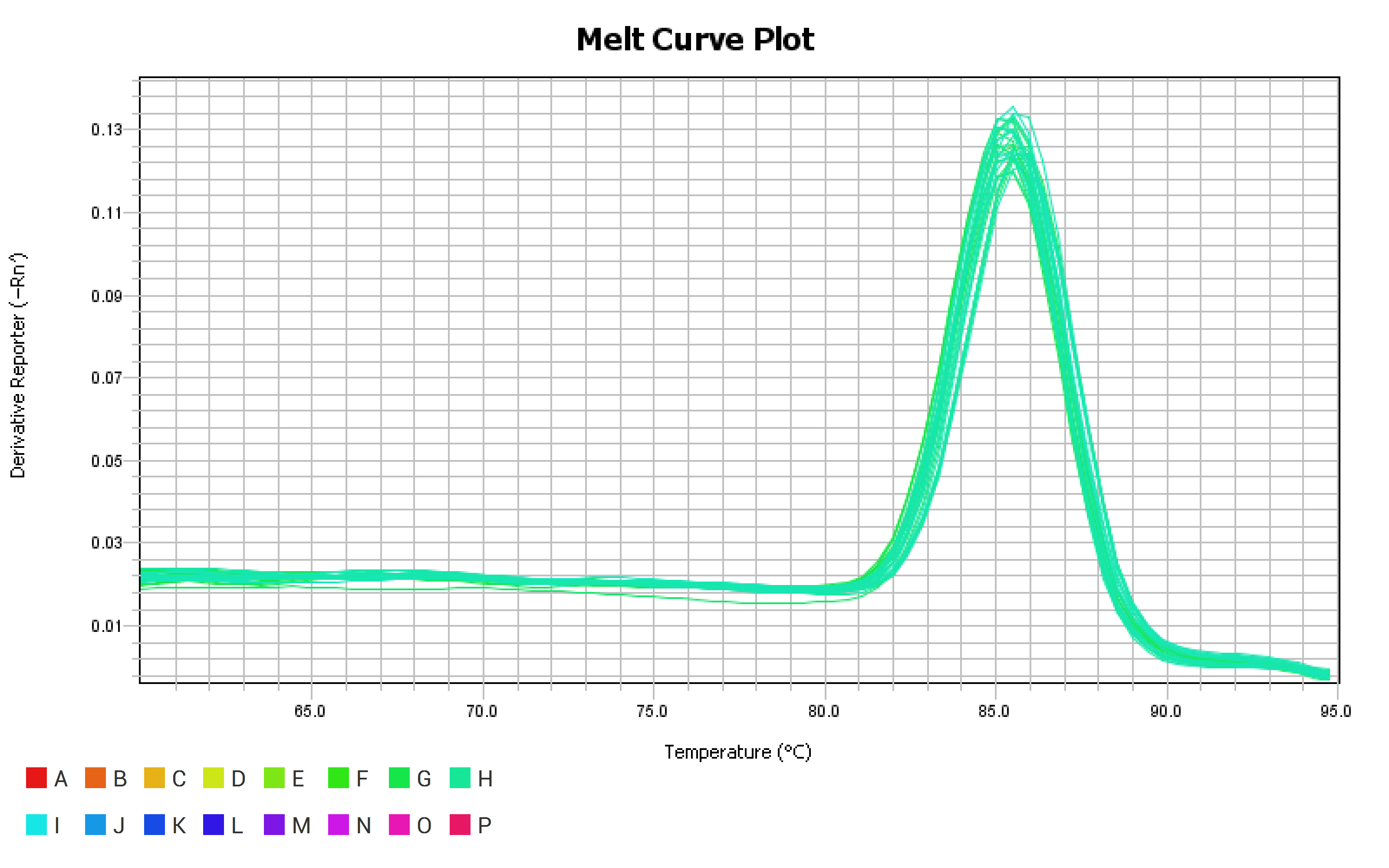

Supplement: Supplemental Information 9 [file peerj-08-9880-s009.zip › raw data-verify/2.RAW DATA-qRT-PCR(HSPA1Aú⌐/4.Melt Curve Plot-qRT-PCR/Melt Curve Plot HSPA1A.jpg]

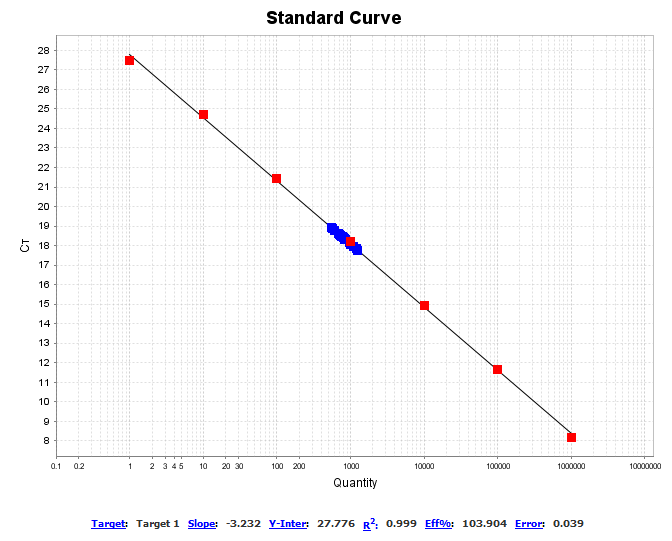

Supplement: Supplemental Information 9 [file peerj-08-9880-s009.zip › raw data-verify/2.RAW DATA-qRT-PCR(HSPA1Aú⌐/5.Standard Curve-qRT-PCR/Standard Curve ACTB.png]

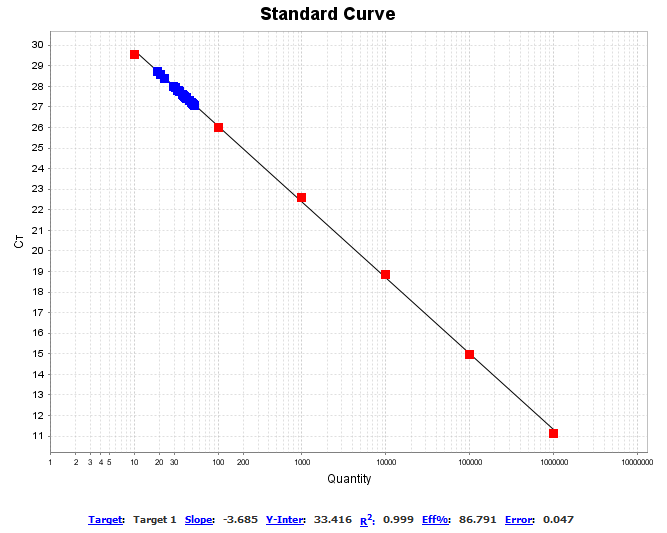

Supplement: Supplemental Information 9 [file peerj-08-9880-s009.zip › raw data-verify/2.RAW DATA-qRT-PCR(HSPA1Aú⌐/5.Standard Curve-qRT-PCR/Standard Curve HSPA1A.png]

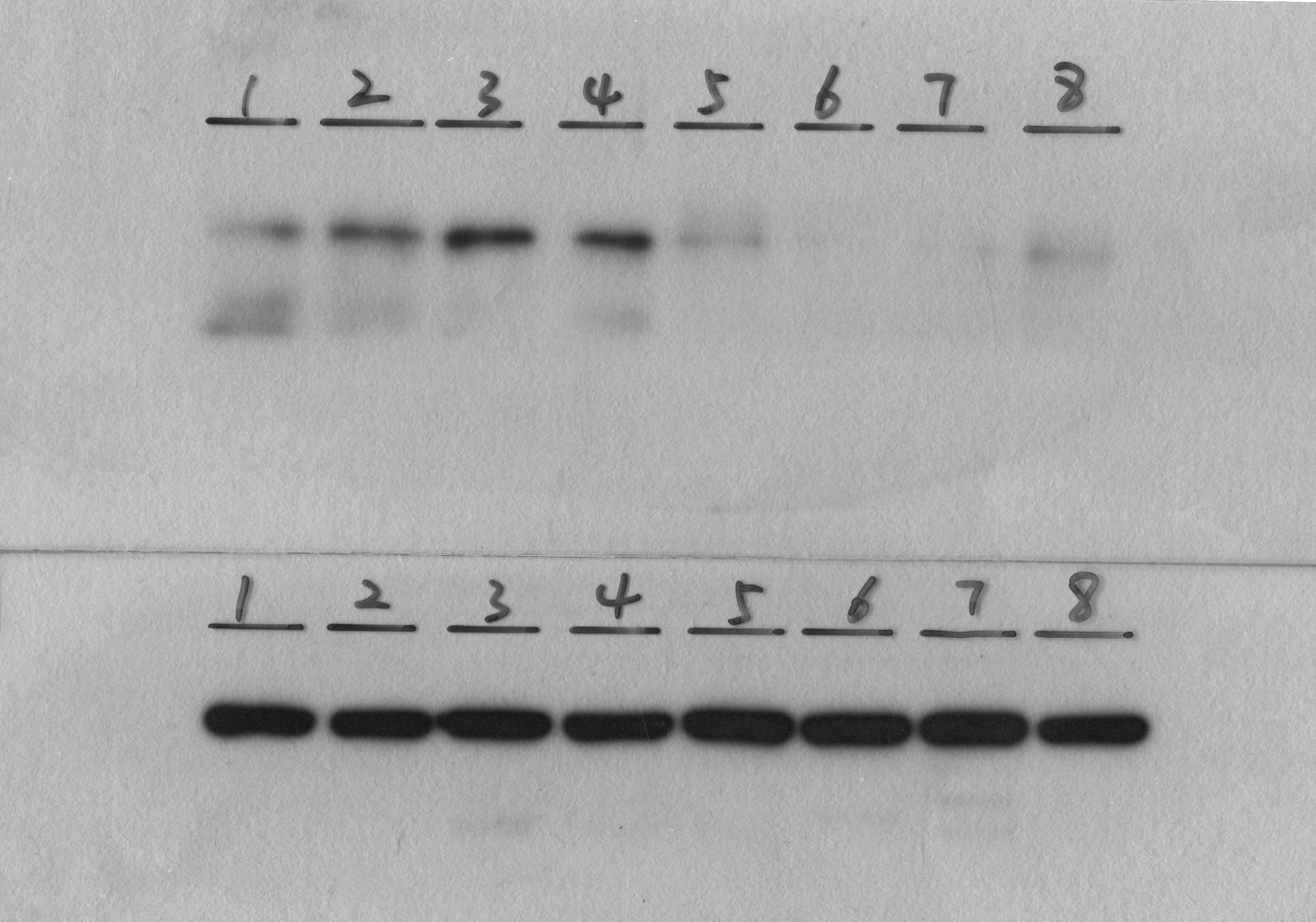

Supplement: Supplemental Information 9 [file peerj-08-9880-s009.zip › raw data-verify/3.Western blot/2.Western blot.tif]
