# Supplementary material for: Integrated analysis of the transcriptome-wide m6A methylome in preeclampsia and healthy control placentas
Source: PeerJ. 2020 Sep 15;8:e9880. doi: 10.7717/peerj.9880 (PMC7500358; doi:10.7717/peerj.9880)
Supplement: Supplemental Information 9 [file peerj-08-9880-s009.zip › raw data-verify/1.RAW DATA-MazF-qPCR/4.Quantifies m6A levels of HSPA1A using MazF-qPCR.doc]

**Quantifies m6A levels of HSPA1A using MazF-qPCR.**

The m6A methylation at that particular site of *HSPA1A* was the expression of HSPA1A with MazF treatment, which was normalized by MazF -:

% MazF - = (2 ^–Ct MazF +^) / (2 ^–Ct MazF -^) * 100%.

*HSPA1A*

| Group | Sample | (MazF +)/ (MazF -) | | |
| --- | --- | --- | --- | --- |
|  |  | (MazF -) (Ct) | (MazF+ )(Ct) | % |
| ECL | ECL001 | 22.217 | 27.823 | 2.053 |
|  | ECL003 | 22.517 | 27.934 | 2.341 |
|  | ECL004 | 22.240 | 27.796 | 2.126 |
|  | ECL005 | 23.341 | 28.138 | 3.598 |
| NEG | NEG002 | 20.705 | 28.588 | 0.424 |
|  | NEG003 | 22.837 | 28.762 | 1.646 |
|  | NEG004 | 22.027 | 29.607 | 0.522 |
|  | NEG005 | 22.637 | 29.238 | 1.031 |
|  | Average ECL |  | | 2.530 |
|  | Average NEG |  |  | 0.906 |
|  | ECL/ NEG |  |  | 2.793 |
|  | p-value |  |  | 0.012 |
